# End-Cretaceous extinction in Antarctica linked to both Deccan volcanism and meteorite impact via climate change

**DOI:** 10.1038/ncomms12079

**Published:** 2016-07-05

**Authors:** Sierra V. Petersen, Andrea Dutton, Kyger C. Lohmann

**Affiliations:** 1 Department of Earth & Environmental Sciences, University of Michigan, 2534 C.C. Little Building, 1100 North University Avenue, Ann Arbor, Michigan 48109, USA; 2 Department of Geological Sciences, University of Florida, 241 Williamson Hall, PO Box 112120, Gainesville, Florida 32611, USA

## Abstract

The cause of the end-Cretaceous (KPg) mass extinction is still debated due to difficulty separating the influences of two closely timed potential causal events: eruption of the Deccan Traps volcanic province and impact of the Chicxulub meteorite. Here we combine published extinction patterns with a new clumped isotope temperature record from a hiatus-free, expanded KPg boundary section from Seymour Island, Antarctica. We document a 7.8±3.3 °C warming synchronous with the onset of Deccan Traps volcanism and a second, smaller warming at the time of meteorite impact. Local warming may have been amplified due to simultaneous disappearance of continental or sea ice. Intra-shell variability indicates a possible reduction in seasonality after Deccan eruptions began, continuing through the meteorite event. Species extinction at Seymour Island occurred in two pulses that coincide with the two observed warming events, directly linking the end-Cretaceous extinction at this site to both volcanic and meteorite events via climate change.

## Abstract (web summary)

The cause of the end-Cretaceous mass extinction is still debated due to difficulties separating the influences of two closely-timed potential causal events (massive volcanism and meteorite impact). Here, the authors link the extinction to both kill mechanisms through a new paleotemperature record.

## Graphical abstract

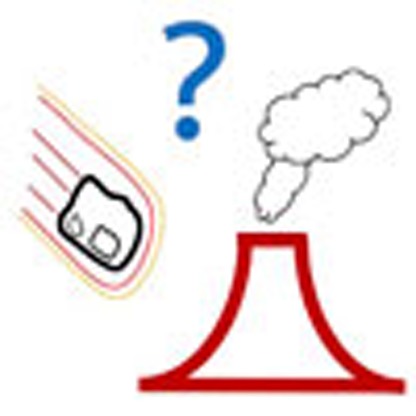

The cause of the Cretaceous–Paleogene (KPg) mass extinction remains controversial due to difficulties in separating the influence of two closely timed potential causal events. Some suggest that the eruption of the massive Deccan Traps volcanic province in India caused species extinction^1^ through trace metal toxicity^2^ or negative effects of volatiles emitted during the eruption (for example, CO_2_, SO_2_)^3^. Others cite the impact of the massive Chicxulub meteorite as the cause^4^,^5^. Recent work has suggested that the impact event might have triggered and accelerated eruption of the Deccan Traps^6^,^7^, further complicating the cause–effect relationship. Distinguishing between the effects of these two potential causal events can be difficult because many KPg boundary (KPB) sites have hiatuses, insufficient temporal resolution, and lack of species continuity across the boundary.

Seymour Island, Antarctica (64°17′ S, 56°45′ W) is particularly well-suited for studying the KPB interval due to its expanded section, continuous sedimentation, and abundant, exceptionally preserved macrofossils, including some species and genera that survive across the KPB^8^,^9^. Previous efforts to link temperature change and biotic turnover at Seymour Island relied on untested assumptions about the value and stability of the oxygen isotopic composition of seawater (δ^18^O_w_) to calculate temperature^10^. These assumptions can now be avoided (and tested) using the carbonate clumped isotope paleothermometer, a new proxy that can measure temperature independent of δ^18^O_w_, and can therefore directly calculate δ^18^O_w_ for each sample^11^.

Here we present a record of high latitude ocean temperature and δ^18^O_w_ from Seymour Island that covers the last few million years of the Cretaceous and crosses the KPB (from ∼69–65.5 Ma), measured on well-preserved bivalve shells. We find evidence of climate change at the onset of Deccan volcanism and again at the KPB that align with two previously identified pulses of extinction^10^, indicating the complete end-Cretaceous extinction at this site is due to the combined effects of the volcanic and meteorite kill mechanisms.

## Results

### Stable and clumped isotope analysis of fossil bivalves

Twenty-nine well-preserved shells, representing five species of bivalve (*Lahillia larseni, Cucullaea antarctica, Cucullaea ellioti, Eselaevitrigonia regina* and *Nordenskjoldia nordenskjoldi*) were selected from the Zinsmeister collection^9^, and were analysed for δ^18^O, δ^13^C, and the clumped isotope composition, Δ_47_ (see Methods). These shells represent the best-preserved individuals from a larger sample set of 116 shells that were analysed for only δ^18^O and δ^13^C (see Methods). Shell preservation was determined by X-ray diffraction, cathodoluminescence and trace element screening (see Methods). δ^18^O_w_ values were calculated for each clumped isotope sample using the Δ_47_-derived temperature, measured δ^18^O of the shell, and an equilibrium temperature relationship for aragonite^12^. Shells come from Units 9 and 10 of the López de Bertodano Formation, which represent an outer to inner shelf environment with a water depth of <200 m (ref. 8). Most shells were sampled in two locations, the umbo (hinge) and the ventral margin (see Methods). An age model was constructed using published magnetostratigraphic data^10^ and was corroborated with published^13^ and new ^87^Sr/^86^Sr measurements (see Methods).

### Cretaceous–Paleogene temperature at Seymour Island

**Figure 1:**
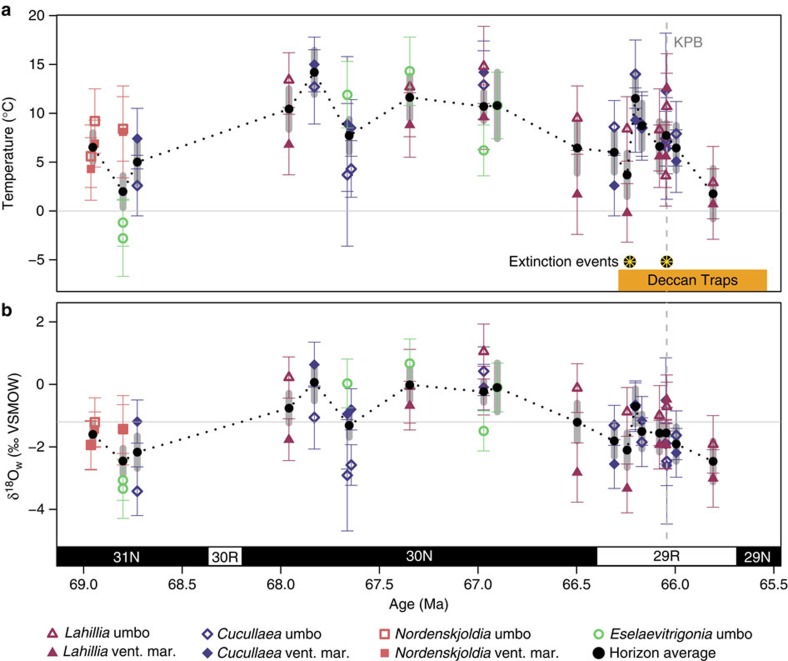
Late Cretaceous temperature and δ^18^O_w_ at Seymour Island. (**a**) Δ_47_-derived temperature and (**b**) δ^18^O_w_ versus age. The timing of Deccan Traps volcanism^7^,^33^ (orange rectangle) and two extinction pulses^10^ (black/orange stars) are shown for comparison. Coloured error bars on individual points represent 1 s.e. of the mean of many replicates or the long-term reproducibility of carbonate standards, whichever is larger (see Methods). Horizon averages in **a**,**b** were calculated as the inverse variance weighted mean, to account for variable reproducibility of samples, with thick grey error bars showing the inverse variance weighted error on the mean (see Methods). Black line connecting horizon means is dashed to represent extrapolation of temperature and δ^18^O_w_ trends between measured horizons. Samples within ±3.5 m stratigraphic position of another sample were combined into a single horizon for horizon means (see Methods). Grey horizontal line in **a** represents the freezing point (0 °C) and in **b** represents the ice free, latitude-adjusted seawater value of −1.2‰ (ref. 30). Vertical grey dashed line denotes the KPg boundary (labelled KPB)^34^. Age model construction is described in Methods. Data for this figure can be found in Supplementary Data 5 and 6.

The Δ_47_-derived temperatures for each species and shell position are shown in Fig. 1a. Temperatures warm from ∼5 °C to ∼14 °C between 68.7 and 67.8 Ma. After ∼1 Myr of sustained warm (∼9–12 °C) temperatures, a gradual cooling back to ∼4 °C occurs from 66.9 to 66.25 Ma. Approximately 150 kyr before the KPg boundary, a marked warming of ∼7.8±3.3 °C occurs. After the initial warming, temperatures decline until a second smaller warming pulse occurs at the KPB (1.1±2.7 °C), followed by continued decline until pre-event levels are reached ∼200–400 kyr after the initial spike. Although the second warming is minor when comparing the horizon means, individual samples reach temperatures near those seen at the peak of the earlier, larger warming spike (Fig. 1a).

The mean ocean temperature for the entire section is 7.7±3.4 °C (1 s.d.), consistent with a terrestrial temperature estimate of 7 °C from fossil wood^14^ and a soil temperature estimate of 10–13 °C from branched tetraethers^15^. Maastrichtian ocean temperatures from Seymour Island have been previously estimated at anywhere from 5 to 16 °C based on δ^18^O of micro- and macro-fossils^10^,^16^,^17^,^18^,^19^, but these estimates assume a fixed δ^18^O_w_ value, which is not appropriate for this location (see below). The long-term temperature pattern is mirrored in all species and shell positions (Fig. 1a; Supplementary Fig. 1), and is robust across different methods of calculating horizon means (see Methods; Supplementary Fig. 2). The pattern is consistent with terrestrial climate reconstructions based on fungal palynomorphs and pollen grains, which document warm, humid conditions during Chron 30 N, bracketed by cooler conditions before and after^20^. Similar pre-KPB warming in Chron 29R, albeit of lower magnitude, has been seen before, with ∼5 °C of warming observed in a terrestrial section in North America^21^ and 2–3 °C of warming recorded in multiple open-marine records^22^,^23^,^24^.

### Patterns in position-specific isotopic and temperature data

**Figure 2:**
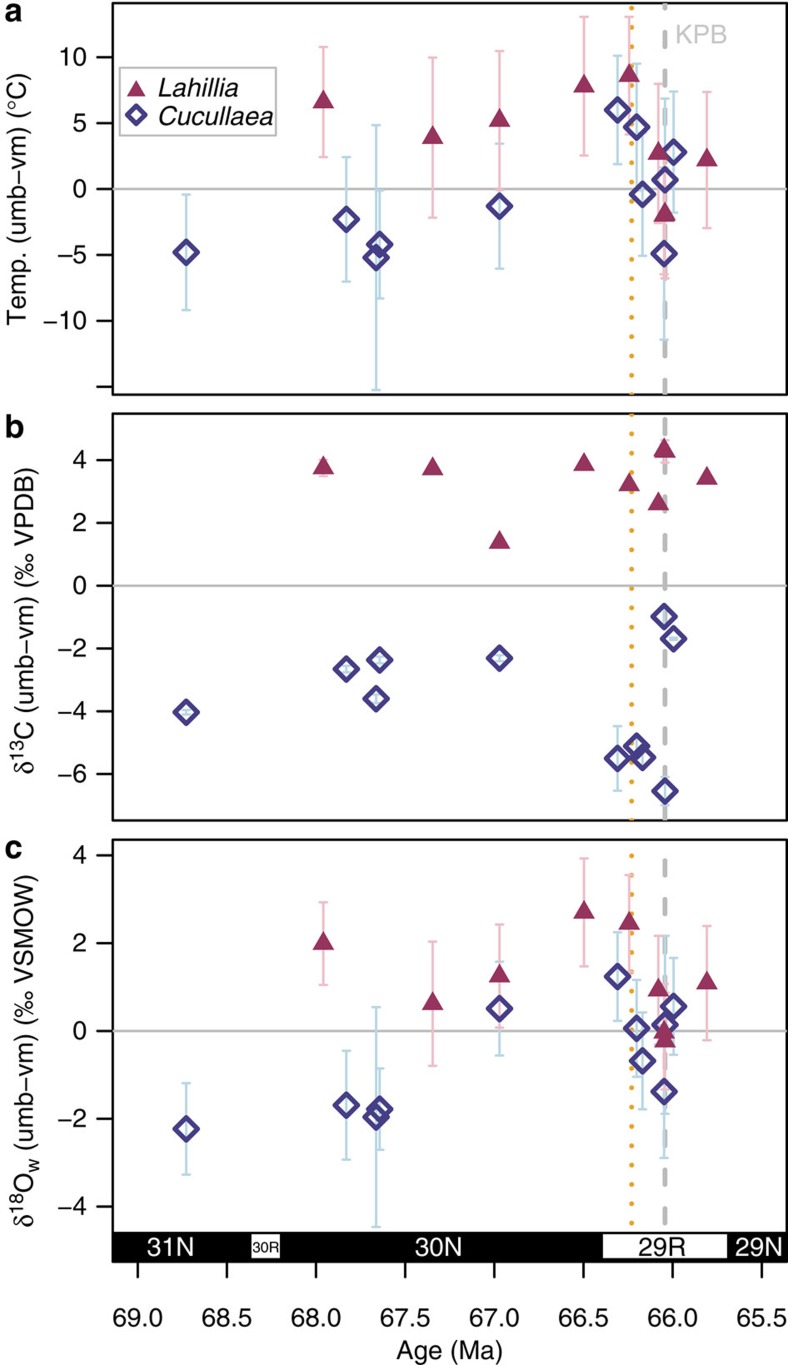
Position-specific differences. Differences in (**a**) temperature, (**b**) δ^13^C and (**c**) δ^18^O_w_ for *Lahillia* and *Cucullaea* versus age. All differences calculated as the umbo minus the ventral margin value (umb-vm), with the horizontal grey line at 0 representing no difference between positions. Error bars are 1 s.e. for temperature and δ^18^O_w_ and 1 s.d. for δ^13^C, propagated through the difference calculation. *Lahillia* consistently shows warmer temperatures, and higher δ^13^C and δ^18^O_w_ in the umbo relative to the ventral margin (+ difference). *Cucullaea* generally shows the opposite, with warmer temperatures, and higher δ^13^C and δ^18^O_w_ in the ventral margin relative to the umbo (– difference), although this pattern is less consistent. After the pre-KPB warming spike, the position-specific differences in temperature and δ^18^O_w_ for both *Lahillia* and *Cucullaea* decrease to within error of zero and remain low until the end of the study interval. Differences in δ^13^C remain fairly constant through the section. Vertical grey dashed line represents the KPg boundary (labelled KPB)^34^ and orange dotted line represents the timing of the first extinction event^10^. Age model construction is described in Methods.

Position-specific measurements of the umbo and ventral margin can provide additional information about seasonal variability. Within a single shell, the difference between the temperature recorded in the umbo and ventral margin can be as great as 7–8 °C or as little as 0 °C. All specimens of *Lahillia* before the warming spike show consistent offsets between the two positions, with temperatures ∼3–9 °C warmer, δ^18^O_w_ values ∼1–3‰ higher, and δ^13^C values ∼1–4‰ higher in the umbo than in the ventral margin (Fig. 2; Supplementary Fig. 3). In contrast, *Cucullaea* specimens have δ^13^C values ∼2–6‰ lower in the umbo than in the ventral margin (Fig. 2b). Position-specific differences in temperature and δ^18^O_w_ for *Cucullaea* are less consistent, but are generally in the opposite direction of those seen in *Lahillia* (Fig. 2; Supplementary Fig. 4).

We suggest these position-specific differences in temperature, δ^18^O_w_, and δ^13^C are due to different seasonal aliasing early and late in the life cycle of the bivalve. Bivalves cease shell growth during reproduction^25^, or when under thermal stress (either too hot^26^ or cold^27^, depending on the taxon), and shell growth slows and becomes more seasonally restricted later in life^28^. For example, Eocene-age *Cucullaea* shells from Seymour Island were shown to cease growth during the warmest months of each year, based on high-resolution δ^18^O measurements^26^. The umbo, which represents early life, should therefore average a larger number of months than the ventral margin, which represents later life when shell growth slows and ceases during reproduction months. For *Lahillia*, the cooler temperature recorded in the ventral margin suggests that shell growth ceased in summer later in life. For *Cucullaea*, the opposite is true, with shell growth biased towards warmer months later in life. The magnitude of the temperature difference between the two positions (0–9 °C) represents a minimum estimate of the seasonal amplitude at this latitude. Position-specific differences can also be seen in δ^18^O and δ^13^C in shells not measured for clumped isotopes (Supplementary Figs 5–7).

### High variability in δ^18^O_w_ within and between shells

**Figure 3:**
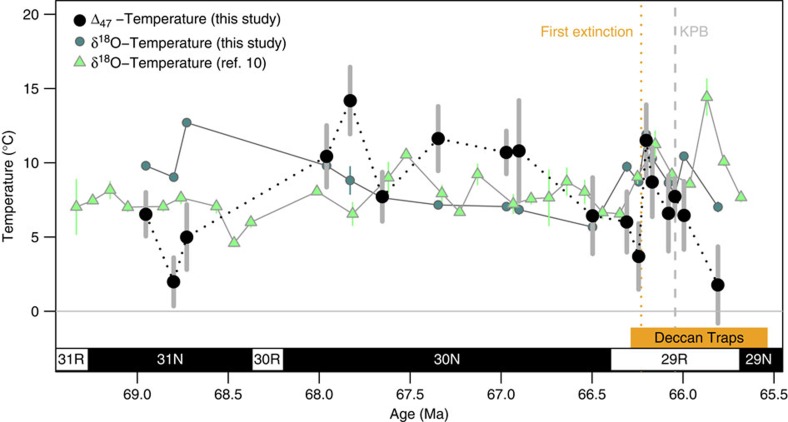
Comparison of Δ_47_-derived and δ^18^O-derived temperature records. Horizon mean Δ_47_-derived temperature shown in black with grey error bars (same as Fig. 1a). Horizon mean was calculated as the inverse variance weighted mean, to account for variable reproducibility of samples, with error bars showing the inverse variance weighted error on the mean (see Methods). Line connecting horizon means is dashed to represent extrapolation of temperature trends between measured horizons. Samples within ±3.5 m stratigraphic position of another sample were combined into a single horizon for horizon means (see Methods). δ^18^O-derived temperature calculated from horizon mean δ^18^O (this study) shown in dark turquoise circles with error bars representing 1 s.d. on δ^18^O, propagated through the δ^18^O-T-δ^18^O_w_ relationship^12^. Published δ^18^O-derived temperature record from Tobin *et al*.^10^, with age model updated to match this study, shown in light green triangles with error bars representing 1 s.e. on the mean of all sample replicates within a given time bin. Both δ^18^O-derived temperature records assume a δ^18^O_w_ value of −1.0‰ (ice-free, globally constant)^29^. If δ^18^O_w_ was instead assumed to be −1.2‰ (ice-free, latitude-adjusted)^30^, the shape of the δ^18^O-derived curves would be identical but shifted ∼1 °C colder. The two δ^18^O-derived temperature curves miss the million-year cool/warm/cool temperature structure, underestimate the magnitude of the pre-KPB warming event and infer an earlier timing for the onset of warming, and do not capture the full range of temperatures seen in the Δ_47_-derived temperature record. Vertical grey dashed line represents the KPg boundary (labelled KPB)^34^ and orange dotted line represents the timing of the first extinction event^10^. Age model construction is described in Methods.

Individual sample δ^18^O_w_ values vary from −3.4 to +1.0‰ through the section, and δ^18^O_w_ is highly correlated to temperature (Fig. 1; Supplementary Fig. 8; Supplementary Discussion). The mean δ^18^O_w_ value for the entire section is −1.3±0.8‰ (1 s.d.). This agrees with the value predicted for an ice-free world (−1.0‰; ref. 29) or for an ice-free world adjusted for the modern latitudinal isotopic gradient (−1.2‰; ref. 30), two δ^18^O_w_ values typically assumed when converting carbonate δ^18^O to temperature. However, the large range and temporal variability in δ^18^O_w_, on the order of ±1–2‰, suggests that the typical assumptions of constant δ^18^O_w_ are incorrect for this location and introduce bias into temperatures calculated from δ^18^O. Figure 3 compares the clumped isotope temperature record to temperature calculated from carbonate δ^18^O for shells from this study and from Tobin *et al*.^10^, assuming a constant δ^18^O_w_ value of −1.0‰. Both δ^18^O-derived temperature records fail to capture the temperature structure seen in the clumped isotope record on both million-year and sub-million-year timescales. For example, both δ^18^O-derived temperature records do not identify the coldest temperatures (<5 °C) around 68.8, 66.4 and 65.7 Ma (Fig. 3). This is because the positively correlated variation in temperature and δ^18^O_w_ have opposite effects on shell δ^18^O, masking both signals and resulting in minimally varying, intermediate δ^18^O values. If a fixed value for δ^18^O_w_ is assumed, the resulting δ^18^O-derived temperature record has only around half the variability of the ‘true' clumped isotope temperature record, which allows for variations in δ^18^O_w_ (7 °C versus 12.5 °C temperature range for shells from this study). Therefore, temperatures derived from δ^18^O of micro- and macro-fossils from Seymour Island that assume a fixed δ^18^O_w_ value^10^,^16^,^17^,^18^,^19^ should only be taken on average, and any time series that do not account for changing δ^18^O_w_ should be treated with caution and assumed to underestimate true temperature variability.

## Discussion

We propose that the observed δ^18^O_w_ variability reflects the influence of continental runoff. Precipitation over land is more depleted in ^18^O than ocean water, sometimes by more than 10–15‰, so any appreciable contribution of runoff would decrease δ^18^O_w_ values in shelf waters. The correlation between δ^18^O_w_ and temperature, with the coldest temperatures correlating with the lowest δ^18^O_w_ values, suggests a climatological control on runoff delivery. Precipitation becomes more depleted in ^18^O as air temperature decreases. In addition, colder temperatures would promote the accumulation of snowpack in winter and the concentrated delivery of isotopically depleted meltwater during the spring melt, reducing δ^18^O_w_ below expected open-marine values. Terrestrial carbon, depleted in δ^13^C relative to marine carbon, could accompany the spring melt, explaining the co-occurrence of low δ^18^O_w_ and low δ^13^C. This hypothesized seasonal meltwater delivery is consistent with the temperature differences observed between positions in *Lahillia* (*Cucullaea*), with lower (higher) δ^18^O_w_ and δ^13^C in the ventral margin, the position skewed more towards colder (warmer) months.

The coldest temperatures recorded in the Seymour Island section (at 68.8, 66.4 and 65.7 Ma) are near the freezing point, implying the possibility of sea ice formation near Seymour Island. Dinoflagellate cyst abundance has previously suggested that sea ice formed at this time^20^. With temperatures near freezing at sea level at the tip of the Antarctic Peninsula, the interior of Antarctica was likely cold enough to sustain year-round glaciers, particularly at high elevation. Global sea level reconstructions record two sea level falls in the late Maastrichtian (KMa4 and KMa5)^31^. Sequence boundaries occur at 66.8 and 68.8 Ma, with sea level minima closely following^31^. The close temporal alignment of sea level lowstands and coldest Antarctic temperatures suggests that the observed sea level fluctuations could be driven by continental ice accumulation on Antarctica.

The ∼8 °C pre-KPB warming observed in the Seymour Island record is larger than similar warming events seen at other marine sites^22^,^23^,^24^. If sea ice or continental ice were present during the coldest interval immediately preceding the warming spike (66.5–66.3 Ma), the disappearance of this ice during warming could accentuate local climate change at Seymour Island through the ice-albedo feedback, the same process that is currently amplifying anthropogenic climate change in the Arctic by roughly a factor of two^32^. In addition, if this warming was accompanied by melting of continental ice, the reduction in global seawater δ^18^O_w_ would mute marine temperature change calculated from δ^18^O assuming constant δ^18^O_w_ (refs 22, 23, 24). One terrestrial record derived from plant assemblages, which are immune to changing δ^18^O_w_ values, indicated a ∼5 °C warming at this time^21^, larger than the 2–3 °C observed at open-marine sites^22^,^23^,^24^. δ^18^O-derived temperature records from Seymour Island^10^ also record a pre-KPB warming of ∼5 °C, but the warming event begins earlier, is more gradual, and is of lower magnitude because this proxy method does not account for concurrent increases in δ^18^O_w_ at this time (Fig. 3).

The majority of Deccan Traps volcanism occurred during Chron 29R, and the oldest formation (Jawhar) has been dated near its base to 66.288±0.027 Ma (ref. 33) and 66.38±0.05 Ma (ref. 34) using two different dating methods. The temporal alignment of the pre-KPB warming event at Seymour Island with the onset of Deccan Traps volcanism suggests a genetic link between volcanic release of CO_2_ and the abrupt increase in temperature. The eruptions that occurred between the onset of Chron 29R volcanism and the KPg boundary emitted anywhere from 270 to 900 p.p.m. CO_2_ (assuming 14 Tg CO_2_ emitted per km^3^ lava erupted^35^, and all CO_2_ remaining in the atmosphere, see Methods) onto a background atmospheric concentration of ∼360–380 p.p.m. (ref. 36). The timing of transient increases in marine carbonate dissolution driven by the emitted CO_2_ suggest the main phase of degassing took place beginning at the onset of Chron 29R, lasting less than 200 kyr (ending before the KPB)^37^. Using the same volume-to-CO_2_ conversion, the remaining (post-KPB) volcanism potentially emitted another 825–900 p.p.m. The magnitude of the second, weaker warming event at the KPB only appears as 1.1 °C in the horizon mean, but individual samples show a potentially larger warming (Fig. 1a). Though the volume of lava erupted (and therefore probably CO_2_ emitted) was larger after the KPB, the climatological effects of these emissions were potentially reduced due to higher background CO_2_ levels and an emissions duration roughly twice as long^7^,^32^. Additionally, the character of eruptions and the geochemical signature of the lava changed at the KPB^6^, which could be correlated with changes in the CO_2_ content and degassing rate. Feedbacks triggered by the meteorite impact may have also played a role in setting temperatures at and after the KPB.

Modelling studies using differing CO_2_ estimates and injection durations predict 0.5–4 °C of global warming^37^,^38^,^39^,^40^. If the sea ice-albedo feedback had the same amplifying impact in Maastrichtian Antarctica as it is having today in the Arctic, the local temperature change could have been as much as double the global value^32^. Roughly 200–400 kyr after the initial large warming event, temperatures at Seymour Island had returned to pre-event levels. This rate of cooling is roughly consistent with the e-folding timescale of silicate weathering (∼240 kyr (ref. 41)), supporting the interpretation that this climate change is the result of changes in atmospheric CO_2_, which would be scrubbed from the atmosphere through this long-term carbon cycle feedback (as well as by other short-term processes).

Species extinction at Seymour Island occurred in two statistically distinguishable pulses^10^, one at the KPg boundary and another one 40 m below, calculated to be at 66.23 Ma in this age model (Fig. 1a). Early studies of Seymour Island also noted a drop in species diversity ∼30 m below the KTB^8^. This earlier extinction pulse coincides with the large warming event seen in our record (66.24–66.20 Ma) and with the onset of Chron 29R Deccan Traps volcanism^7^,^33^. The two extinction intervals are of similar magnitude (14 and 10 species eliminated, respectively), but differ in their extinction patterns, suggesting different kill mechanisms. In the earlier (Deccan) extinction, 9 of 10 species were benthic, whereas in the second (Chicxulub, KPB) extinction, only 6 of 14 species were benthic^10^. Only one ammonite species became extinct in the first event, whereas all remaining (six) ammonite species became extinct at the KPg boundary^10^,^42^.

The warm temperatures reached at the peak of warming alone could not have caused the first pulse of extinction. Many of the affected species were also present during Chron 30N (ref. 10), when temperatures were equivalently warm. Instead, it is possible that the rate of warming, as opposed to the maximum temperatures reached, led to extinction. The rate of warming during the Deccan temperature spike appears to be more rapid than at any other temperature change seen in the record (Fig. 1a), although this may be an artifact of the variable sampling resolution. Other indirect effects of volcanic emissions could also have played a role in the extinction, such as trace metal toxicity^2^, ocean acidification from increased atmospheric CO_2_, or short-term cooling and acid rain from emissions of SO_2_ (ref. 3).

The lowest δ^13^C values are observed in the interval between the pre-KPB and KPB events. Low δ^13^C values have been measured at the KPB from and nearby Seymour Island before^16^,^43^, which could be an indication of a reduction in surface ocean productivity, as has been previously suggested^44^,^45^,^46^,^47^. The difference in temperature between the umbo and ventral margin in *Lahillia*, a minimum estimate of seasonality, decreased from 5-8 °C to near 0 °C after the pre-KPB warming pulse and remained low until the end of the study interval. *Cucullaea* also shows little to no temperature difference between shell positions after the pre-KPB warming event. This could indicate a reduction in seasonality or a reduced growing season due to environmental stresses occurring after the onset of volcanism.

Roughly half of the total species extinction at Seymour Island (10 of 24 species) occurred before the Chicxulub impact event and can be temporally and climatologically linked to Deccan Traps volcanism, indicating the volcanic kill mechanism definitively contributed to the end-Cretaceous extinction. The remaining extinctions (14 of 24 species) took place at the KPg boundary, at the time of the Chicxulub impact, but also the beginning of the post-KPB phase of Deccan volcanism, which was potentially triggered and/or accelerated by the impact itself^6^,^7^. It is therefore impossible at this point to separate the influences of the meteorite and the additional volcanism in driving the second phase of extinction or in causing the second warming pulse. Nevertheless, the full magnitude of the end-Cretaceous extinction at this site can be attributed to the combined influence of Deccan Traps volcanism and the meteorite impact event, previously described as a press-pulse extinction mechanism^48^,^49^. Importantly, the pre-KPB warming, which itself correlated with significant extinction, may have increased ecosystem stress, making the ecosystem more vulnerable to collapse when the meteorite hit. This sequence of events may have combined into a ‘one-two punch' that produced one of the largest mass extinctions in Earth history.

## Methods

### Environment and sample selection

Samples are from the W.J. Zinsmeister collection, formerly housed at Purdue University, and now located at the Paleontological Research Institute in Ithaca, NY, USA. Specimens were collected from Seymour Island from the López de Bertodano and Sobral Formations, covering the Late Cretaceous to early Paleocene. The stratigraphic framework used in this study is described by Zinsmeister *et al*.^50^ and has a stratigraphic resolution of ±3.5 m. Samples come from Units 9 and 10 of the López de Bertodano Formation, part of the upper beds (Units 7–10, Molluscan Units), as defined by Macellari^8^. The Molluscan Units contain diverse and abundant bivalve, gastropod, and ammonite macrofauna. Since the Cretaceous, these sediments are thought to have experienced only mild diagenetic conditions^51^. The depositional environment is interpreted to be inner to outer shelf, a semi-enclosed or quiet environment, with water depths less than 200 m, on the basis of faunal assemblages, grain size and sorting analysis, diversity of macrofauna, and abundance of glauconite^8^. Interpretations of water depth variation through the López de Bertodano Fm. disagree, with some suggesting a shallowing-upward^52^ and others a deepening-upward^8^ trend through the section. Overall, the homogeneous nature of the Molluscan Units 7–9 suggests^8^,^42^,^52^, at most, minor variation in water depth over this interval. Although there is some evidence of methane seeps on Seymour and the surrounding islands, it is restricted to lower in the stratigraphy (Units 1–6)^53^. There is no indication of methane seeps in the upper López de Bertodano Formation on Seymour Island^43^.

Bivalve shells (116) were selected from the Zinsmeister collections at Purdue for use in the PhD thesis of author Andrea Dutton^54^ with the aim of including as many stratigraphic horizons as possible for maximum temporal resolution (the ‘full sample set'). Samples were selected from six species (*L. larseni, C. antarctica, C. ellioti, E. regina*, *D. drygalskiana* and *N. nordenskjoldi*) to provide internal consistency between horizons. All these species were thought to live in shallow waters and be infaunal suspension feeders, with the exception of *Nordenskjoldia*, which is thought to be an epibyssate suspension feeder^8^.

A subset of 29 shells were selected from the full sample set for clumped isotope analysis, representing five of the six species in the original study (eliminating *D. drygalskiana*; the ‘clumped isotope sample set'). These were again selected for maximum stratigraphic coverage and species overlap, but were filtered to only include the best-preserved samples, as indicated by low trace element concentrations (see below). A list of samples in the full sample set and those sub-selected for clumped isotope analysis can be found in Supplementary Data 1.

### Sample preparation and extraction

Bulk samples of 50–100 μg were drilled from the umbo, mid-shell and ventral margin of all shells in the full sample set for stable isotope and trace element analysis. Samples were drilled to average over multiple growth bands to represent typical mean annual conditions. The umbo position was centred near the cusp of the umbo. The ventral margin (‘back') was within 1 cm of the edge of the shell. The mid-shell position was half way between the umbo and ventral margin. In the case of broken, partial shells, it was not always possible to sample the ventral margin and/or mid-shell position. Broken and partial shells were not selected for the clumped isotope sample set.

The 29 shells selected for clumped isotope analysis were re-drilled in two positions, the umbo and ventral margin. Sampling locations were directly adjacent to earlier drill locations. Sample size requirements for clumped isotope analysis are much larger than for stable isotope analysis^11^, so 20–30 mg of sample material was collected at each position and homogenized. During drilling for clumped isotope analysis, the drill was operated at the lowest possible rotation speed (1,000 r.p.m.) and pressure was only applied for a few seconds at a time to minimize frictional heating. Based on tests with other samples in our lab, at this drill speed, we do not expect to see any resetting effects. Most shells were drilled from the exterior at a position that was clear of external encrustation or fractures. In a few cases, shells were cut in half and were drilled on the cut interior surface. No correlation was seen between measured values and interior versus exterior drilling. Six shells of the genus *Eselaevitrigonia* were only sampled in the umbo position because invariant stable isotopic compositions between positions suggested that identical environmental conditions were being recorded at all points on the shell (see below), and some shells were broken such that the ventral margin was no longer present.

### Trace element analysis

Elemental analysis of Sr, Mg, Ca, Fe and Mn was carried out on powdered splits of all bulk samples using either an inductively coupled plasma optical emission spectrophotometer (ICP-OES) or an inductively coupled plasma mass spectrometer (HRICP-MS) depending upon the amount of powdered carbonate available for analysis. Analysis was performed in the Keck Elemental Geochemistry Laboratory at the University of Michigan (Director: K.C. Lohmann). Smaller samples were run on HRICP-MS owing to the lower detection limits attainable on this instrument. By comparison with lab standards and of replicate analyses, precision of both types of analyses was maintained at better than 3%. Because these samples were too small to allow for efficient weight determination, calculation of cation concentrations within the carbonate was achieved by assuming that Sr, Mg, Ca, Fe, Mn are carbonate-bound cations and that these five elements account for 100% of the carbonate-bound cations. Trace element data can be found in Supplementary Data 1.

### Assessment of shell preservation

Many lines of evidence (lack of smectite-illite transition, mineralogical and vitrinite-reflectance data) suggest a maximum burial depth of less than 1 km and a maximum burial temperatures of less than 80 °C for the López de Bertodano Formation^51^,^55^. Visual inspection shows shell preservation is excellent, with many shells still displaying pristine mother-of-pearl sheen, indicating the presence of primary aragonite. This was confirmed by conducting X-ray diffraction of powdered material from seven shells representing each of the six species in the full sample set, taken from across the full range of the stratigraphic section. These were shell numbers C1556E (*C. antarctica*), L776E (*L. larseni*), D1110I (*D. dryganskalia*), E1109E (*E. regina*), N1614C (*N. nordenskjoldia*), L1161A2 (*L. larseni*) and C1430 (*C. ellioti*). In all cases, the mineralogy was completely aragonitic and there was no indication of partial conversion to calcite.

Thick or thin sections were made for nine shells (sample numbers C757C2, L757B, L1480B, L1480E, C1467A, L1529A, C915A2, L1430A1, and L1609). Primary textures, including excellent preservation of growth band structures, were observed in shell carbonate from these sections (Supplementary Fig. 9). These sections were also viewed under cathodoluminescence, which can detect elevated amounts of Mn^2+^ that may become incorporated in diagenetic carbonate due to post-depositional water-rock interaction. Shells were non-luminescent, except in rare instances where fractures cut across the shell. This observation is particularly notable because it indicates that pore- fluids in the host sediment were reducing and enriched in Mn^2+^, yet they did not affect bulk shell chemistry. Therefore, alteration is likely only a concern in areas where the shells are fractured. Fractured areas were avoided in sample extraction.

Shell preservation was also assessed through trace element composition. Acceptable levels of Fe and Mn for a well-preserved shell have been determined differently by different authors^17^,^56^. Conservative trace element thresholds of 500 p.p.m. for Fe and 200 p.p.m. for Mn were set, and samples with concentrations above these were deemed ‘potentially altered' and were excluded from further analysis. Of 339 total bulk samples, 42 individual bulk samples (representing 32 unique bivalve shells) had elevated trace element concentrations, indicating overall excellent preservation of samples at Seymour Island.

Shells selected for clumped isotope analysis all had Fe and Mn concentrations below the defined thresholds in the umbo and ventral margin areas. In three cases (N1416B, C772A and C1109A), the trace element composition was only measured at one of the positions on the shell but both positions were sampled for clumped isotopes. In three other cases (C757C-umbo, C915A1-umbo and C915A1-back), the trace element measurements came from the opposite half of the bisected shell, in a mirror image position to where the shell was sampled for clumped isotope analysis. For two shells (L757 and L1430A1), the trace element samples were taken from the outside of the shell, whereas the clumped isotope samples were taken from the inside after the shell was cut in half.

### Stable and clumped isotope analysis

Stable carbon and oxygen isotope data were generated for all bulk samples from the full sample set using an automated Kiel device coupled to a Finnigan MAT 251 isotope ratio mass spectrometer in the University of Michigan Stable Isotope Laboratory (Director: K.C. Lohmann). Before reaction with phosphoric acid in the Kiel device, individual powdered carbonate samples were first roasted in vacuo at 200 °C for 1 h to remove volatile contaminants. Precision of the data was maintained at better than 0.1‰ by calibration to carbonate standards (both internal lab standards and NBS-19), measured daily. All carbonate stable isotope compositions are reported relative to Vienna Peedee Belemnite (VPDB). Stable isotope data for bulk samples can be found in Supplementary Data 1 and are shown in Supplementary Figs 5–7.

Samples in the ‘clumped isotope sample set' were measured for clumped isotopic composition at the University of Michigan Stable Isotope Laboratory. Carbonate powders were prepared on an offline sample preparation device described by Defliese *et al*.^57^. Samples measured in 2015 were cleaned using the ‘WarmPPQ' configuration, whereas samples measured in 2014 used the ‘ColdPPQ' configuration, and were corrected accordingly for fractionations introduced by the Porapak trap^58^.

Standard gases heated to 1,000 °C and equilibrated with water at 25 °C were measured alongside samples and were used to define the absolute reference frame^59^. An acid fractionation factor of 0.067‰ was used, chosen to reflect the 75 °C reaction temperature^57^. Two carbonate standard materials, Carrara Marble and Joulter's Cay Ooids, were measured alongside samples. Accepted Δ_47_ values for these two standards are 0.414‰ and 0.704‰, respectively^57^,^58^. Over the three measurement sessions presented here, the mean values for Carrara and Ooids were 0.423±0.005‰ (1 s.e., *n*=15) and 0.712±0.007‰ (1 s.e., *n*=13). Raw voltages are converted into delta values following Huntington *et al*.^60^, including the same defined values for *λ* and for the isotopic composition of VPDB and VSMOW, and Δ_47_ values were converted to temperature using equation 6 of Defliese *et al*.^57^.

The stable isotopic composition of the powders in the ‘clumped isotope sample set' was measured simultaneously with the measurement of the clumped isotopic composition. Oxygen isotope data were corrected for fractionation during acid digestion using an acid fractionation factor of 1.00836 for aragonitic samples, determined empirically using the aragonitic Ooids carbonate standard, separately calibrated to NBS 18 and NBS-19 (ref. 58). Oxygen isotopic composition of water was calculated using the aragonite-water fractionation of Kim *et al*.^12^, and is reported relative to VSMOW. Measured clumped isotopic and stable isotopic compositions of ‘clumped isotope sample set' shells can be found in Supplementary Data 2, 3, 4, 5. Carbonate and gas standard data can be found in Supplementary Data 2, 3, 4.

### Calculation of sample and horizon averages

Average δ^13^C and δ^18^O values are calculated as the mean of many (*n*=2–5) replicates, with error taken as 1 s.d. Average values for Δ_47_, temperature, and δ^18^O_w_ are taken as the mean of *n* replicates with the error taken as 1 s.e. on the mean, as is typical in the clumped isotope community (Supplementary Data 5). δ^18^O_w_ is first calculated individually for each replicate from each δ^18^O and Δ_47_-derived temperature pair, then mean δ^18^O_w_ is taken as the mean of many replicates, with 1 s.e. error. In some cases, the calculated 1 s.e. is smaller than the long-term performance of our carbonate standards. We determine ‘external error' on Δ_47_ for a given sample to be the larger of (1) the calculated 1 s.e. of the mean of *n* replicates or (2) the standard deviation of all replicates of Carrara (0.019‰) divided by the square-root of *n* replicates (Supplementary Data 5). For two to five replicates, this gives a 1 s.e. of 0.013‰, 0.011‰, 0.009‰ and 0.008‰. The ‘external error' on temperature is calculated as half of *T*(mean Δ_47_−extSE.)−*T*(mean Δ_47_+extSE), where *T*(*x*) is the Δ_47_−Temperature calibration function and ‘extSE' is the external s.e. replacing the calculated s.e. Based on typical 1 s.e. values on δ^18^O_w_ for a sample with a Δ_47_-error of 0.013‰, 0.011‰, 0.009‰ and 0.008‰, the ‘external error' on δ^18^O_w_ is assigned to be 0.92‰, 0.78‰, 0.64‰ and 0.57‰ for *n*=2–5 replicates, respectively. External error is used in all figures and calculations for Supplementary Data 6, 7, 8.

Most (11 of 18) horizons are represented by two samples taken from different positions on a single shell. Where more than one shell is combined (6 of 18 horizons), all samples are treated equally, regardless of position or shell. Only one horizon is represented by a single position on a single shell (*E. regina* at 66.9 Ma). Due to consistently observed patterns within species, we interpret differences between shell positions to reflect environmental aliasing, not scatter around a mean value in a noisy data set. Therefore, the error calculated on a horizon average does not represent certainty on the average value of a noisy data set, but instead represents the spread in data from different shell positions, possibly an indicator of seasonality, combined with some degree of measurement noise. We do not know which species or shell position best represents mean annual temperature, so we felt combining all points equally was the fairest way to treat the data.

Horizon means (Supplementary Data 6, 7, 8) are calculated using equation (1) as the inverse variance weighted mean of all samples from a given horizon, combining samples within the uncertainty of the stratigraphy (±3.5 m)^50^.


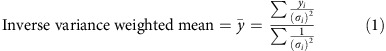


Error on the horizon mean is calculated using equation (2) as error on an inverse variance weighted mean, to maintain consistency.


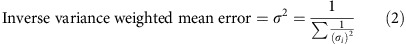


Inverse variance weighted mean was selected to account for the variable reproducibility of samples and not overweight the mean towards a poorly reproducing sample (for example, Sample C1109A at 67.7 Ma). Supplementary Fig. 2 compares multiple methods for calculating horizon mean (for example, inverse variance weighted mean, traditional mean, average of shell averages). The use of traditional mean versus inverse variance weighted mean does not change the long-term pattern, interpretations, or conclusions. The largest difference is seen around 67.7 Ma where there are two poorly reproducing samples.

Taking the average of positions within each shell before combining shells is mathematically equivalent to averaging all points if using the inverse variance weighted mean. Averaging within-shell first only affects four horizons mean minimally at that, if using the traditional mean. We chose to combine adjacent samples based on stratigraphic position, but a composite record could also have been created by combining samples into equal-time bins. Equal-time bins combine samples from before and after the warming spike, resulting in a ‘smoothed' temperature record. If the bin size is small enough (50 kyr), two warming pulses are still observed.

### Age model construction

To construct an age model using the recently published magnetostratigraphic data from Seymour Island^10^, the stratigraphic heights of chron reversals were converted to the Zinsmeister stratigraphic framework^50^ based on the established position of the KPg boundary in each (1,059 m for this study, 865 for Tobin *et al*.^10^) and assuming equal unit thickness. The stratigraphic height of a point in the Zinsmeister framework is therefore calculated as the height in the Tobin framework plus the difference in the KPg boundary positions (1,059–865=194 m). This assumption is supported by the position of the Unit 8–Unit 9 boundary, which is at 652 m in the Tobin framework^10^. The age model conversion would predict that this Unit boundary should be found at 846 m in the Zinsmeister framework, and it is described as ∼850 m by Zinsmeister^50^. Based on this close agreement, when comparing extinction events observed in the Tobin framework with climatic events from this study, any events occurring between the base of Unit 8 and the KPB should be in agreement to within ∼4 m.

The ages of the relevant chron reversals were taken from the most recent Geological Time Scale (2012)^61^, and the age the KPg boundary was taken as 66.043±0.043 Ma (ref. 34). The position of chron reversals was taken as the average of the stratigraphic heights of the first sample listed above and below the reversal position^10^. This is different from the age model used by Tobin *et al*.^10^, which used the 2004 edition of the Geologic Time Scale with the age of the KPg boundary at 65.5 Ma. To plot the δ^18^O-derived temperature record from Tobin *et al*.^10^ (Fig. 2), ages for chron reversals were updated to the 2012 Geological Time Scale^61^.

Sample ages were linearly interpolated between age model tie points, listed in Supplementary Table 1. There is inherent uncertainty in magnetostratigraphy-based age models due to this interpolation and uncertainties in the construction of the Geological Time Scale^61^. Although not explicitly accounted for, this uncertainty should be considered when comparing events in our age model with absolute U-Pb or ^40^Ar/^39^Ar ages for the Deccan Traps^7^,^33^.

### Strontium isotope analysis

Ten shells were analysed for strontium isotopes for use in corroborating the age model construction. Analyses were performed in the Biogeochemistry and Environmental Isotope Geochemistry Laboratory at the University of Michigan (Director: J.D. Blum). Powdered samples of bivalve aragonite were dissolved in nitric acid and strontium was subsequently separated using ion-exchange chromatography with Eichrom Sr-specific resin. The effluent was dried and loaded onto a tungsten filament for isotope ratio analysis using a Finnigan MAT 262 thermal ionization mass spectrometer. The ^86^Sr/^88^Sr ratio was normalized to 0.1194 and measured ^87^Sr/^86^Sr ratios were corrected for the difference between the long-term laboratory average of NIST 987 (0.710252±0.000026 (2*σ*)) and the accepted value of 0.710248. Analytical uncertainty was calculated using the standard deviation of NIST 987 over a 12-month period (2*σ*=0.000026).

One sample (L1480B) produced a strontium isotopic composition that is not possible to match to the LOWESS curve at any point between 65 and 69 Ma. L1480B also displays anomalously high Sr/Ca values (10–20 mmol mol^−1^), suggesting that it may have been diagenetically altered. Another sample (L1480E) from the same locality produced a reasonable strontium isotope value, so L1480B was ignored. L1480B was also avoided for clumped isotope analysis. Strontium isotope data measured in this study can be found in Supplementary Table 2.

### Corroboration of age model with Sr-isotope data

Strontium isotopic data was not used in the creation of the age model described above. These data can, therefore, be used to assess the validity of the age model constructed based on magnetostratigraphy and stratigraphic correlation. In addition to new strontium isotope measurements made on bivalve samples from this study (Supplementary Table 2), strontium isotope data from other Maastrichtian bivalves from the Zinsmeister collection have been previously published (Supplementary Table 3)^13^. Because they come from the same collection, their stratigraphic positions can be directly compared. Ages were calculated for all published samples using the age model described above based on their stratigraphic position in the Zinsmeister stratigraphic framework^50^. Supplementary Fig. 10 shows a comparison of measured ^87^Sr/^86^Sr values and calculated ages with the most recent seawater strontium isotope curve (LOWESS 5)^62^. There is good agreement between the bivalve data and the seawater curve, lending strong credence to the magnetostratigraphic age model and the stratigraphic conversion between the Zinsmeister framework and the Tobin framework.

### Stable isotope trends in the full sample set

In the paper we discuss systematic differences in δ^13^C, temperature, and δ^18^O_w_ between the umbo and back position seen in samples in the clumped isotope sample set (Figs 1 and 2; Supplementary Figs 3,4 and 11). Supplementary Fig. 11 also shows the relationship between position and shell δ^18^O. Systematic position-specific behaviour in δ^18^O is complicated due to the competing influences of δ^18^O_w_ and temperature, which vary together in a way to oppositely influence δ^18^O (Supplementary Fig. 8). Despite this, *Cucullaea* still consistently shows higher δ^18^O in the ventral margin position, and *Lahillia* generally shows higher δ^18^O in the umbo position.

Although we cannot look at trends in temperature and δ^18^O_w_ in the full sample set, we can still look at variation in δ^13^C and δ^18^O. In the full sample set, *Lahillia* and *Cucullaea* maintain the position-specific relationships in δ^13^C and δ^18^O seen in the limited clumped isotope sample set (Supplementary Figs 5–7). *Cucullaea* has the highest δ^13^C and δ^18^O values in the ventral margin, whereas *Lahillia* has the highest δ^13^C and δ^18^O values in the umbo. The consistency in position-specific relationships in the larger data set suggests that the same position-specific behaviour in temperature and δ^18^O_w_ is likely present in all samples, even if not measured for clumped isotopes.

*Nordenskjoldia* and *Dozyia* show similar position-specific relationships in δ^13^C and δ^18^O to *Cucullaea* and *Lahillia*, respectively, but of lower total magnitude. *Eselaevitrigonia* shows a much smaller variation in δ^18^O than *Cucullaea* or *Lahillia* and δ^18^O does not correlate to δ^13^C as in the other species. This lower variability in stable isotopes suggests consistent environmental and growth conditions between early and late life for this species. For this reason, *Eselaevitrigonia* was only sampled in the umbo position. This decision is supported by the small differences in temperature and δ^18^O_w_ seen in *Nordenskjolia*, another species showing small variations in stable isotopes (Fig. 1).

### Calculations of CO_2_ emissions from Deccan Traps volcanism

Richards *et al*.^6^ tentatively place the KPB between the Bushe and Poladpur formations in the Deccan volcanic sequence due to evidence for a hiatus in volcanism and fracturing and faulting in the lower Bushe formation that did not extend into the higher Poladpur formation. This placement is consistent with existing dating of the Deccan Traps, which unfortunately has a sampling gap in the critical region preventing direct placement of the KPB^7^,^33^. Assuming this stratigraphic placement of the KPB, we determined an estimate of the minimum and maximum volume of lava erupted between the onset of Chron 29R Deccan volcanism and the KPB, which includes all formations between (and including) Jawhar and Bushe (Kalsubai and Lonavala subgroups).

Estimates of the volume of lava erupted in this interval range from 150 × 10^3^ km^3^ (ref. 6) to 500 × 10^3^ km^3^ (ref. 35). Using an emission rate of 14 Tg CO_2_ km^−3^ lava^34^, a modern atmospheric mass of 5.15 × 10^21^ g and an atmospheric density of 28.966 g mol^−1^ air, these lava volumes equate to 270–900 p.p.m. CO_2_ emitted to the atmosphere. Compare this to end-Maastrichtian atmospheric CO_2_ levels, which are thought to be ∼360–380 p.p.m. CO_2_ (ref. 36). The exact timing of lava extrusion during this interval is unknown, but a lack of red boles in the older portion of the section implies very few hiatuses^7^,^63^. The transient timing of marine carbonate dissolution suggests the main phase of degassing occurred beginning shortly after the onset of Chron 29R and ending within 200 kyr (ref. 37).

We also estimate CO_2_ emissions during the interval between the KPB and the end of Chron 29R volcanism, which includes the Poladpur to Mahabaleshwar formations (Wai subgroup) based on our hypothetical placement of the KPB. The post-KPB portion of Deccan volcanism emitted another 461 × 10^3^ km^3^ (ref. 6) to 500 × 10^3^ km^3^ (ref. 35) of lava, equivalent to 830–900 p.p.m. CO_2_ added to the atmosphere. The Wai subgroup eruptions contain more red boles^7^,^63^, suggesting hiatuses between eruptive events. Based on published dates^7^,^33^, the duration of the post-KPB eruptions is roughly twice the length of the pre-KPB portion (∼500 kyr versus 250 kyr), although this does not account for potential hiatuses within the eruptive sequence. Although the total volume of lava erupted (and therefore CO_2_ emitted, based on our assumptions) was larger post-KPB, the climatological impacts might have been smaller due to higher background CO_2_ levels and a longer duration of eruption. Additionally, the emission rate of 14 Tg CO_2_ km^−3^ lava^35^ is an average value. Changes in the character of eruptions and the geochemical signature of the lava before and after the Bushe/Poladpur contact (the hypothesized KPB)^6^ could coincide with changes in the lava's CO_2_ content and therefore emission rate.

### Data availability

The data shown and discussed in this paper is presented in full in the Supplementary Material.

## Additional information

**How to cite this article**: Petersen, S. V. *et al*. End-Cretaceous extinction in Antarctica linked to both Deccan volcanism and meteorite impact via climate change. *Nat. Commun.* 7:12079 doi: 10.1038/ncomms12079 (2016).

## Supplementary Material

Supplementary Information Supplementary Figures 1-11, Supplementary Tables 1-3, Supplementary Discussion and Supplementary References.

Supplementary Data 1 Stable and Trace Element Data for full sample set

Supplementary Data 2 Raw clumped isotope data - September 2014 measurement session

Supplementary Data 3 Raw clumped isotope data - January 2015 measurement session

Supplementary Data 4 Raw clumped isotope data - June 2015 measurement session

Supplementary Data 5 Sample average clumped isotope data.

Supplementary Data 6 Horizon mean temperature, calculated multiple ways

Supplementary Data 7 Composite temperature record, calculated using 50kyr equal time bins

Supplementary Data 8 Composite temperature record, calculated using 100kyr equal time bins
