# Supplementary material for: End-Cretaceous extinction in Antarctica linked to both Deccan volcanism and meteorite impact via climate change
Source: Nat Commun. 2016 Jul 5;7:12079. doi: 10.1038/ncomms12079 (PMC4935969; doi:10.1038/ncomms12079)
Supplement: Supplementary Information — Supplementary Figures 1-11, Supplementary Tables 1-3, Supplementary Discussion and Supplementary References. [file ncomms12079-s1.pdf]

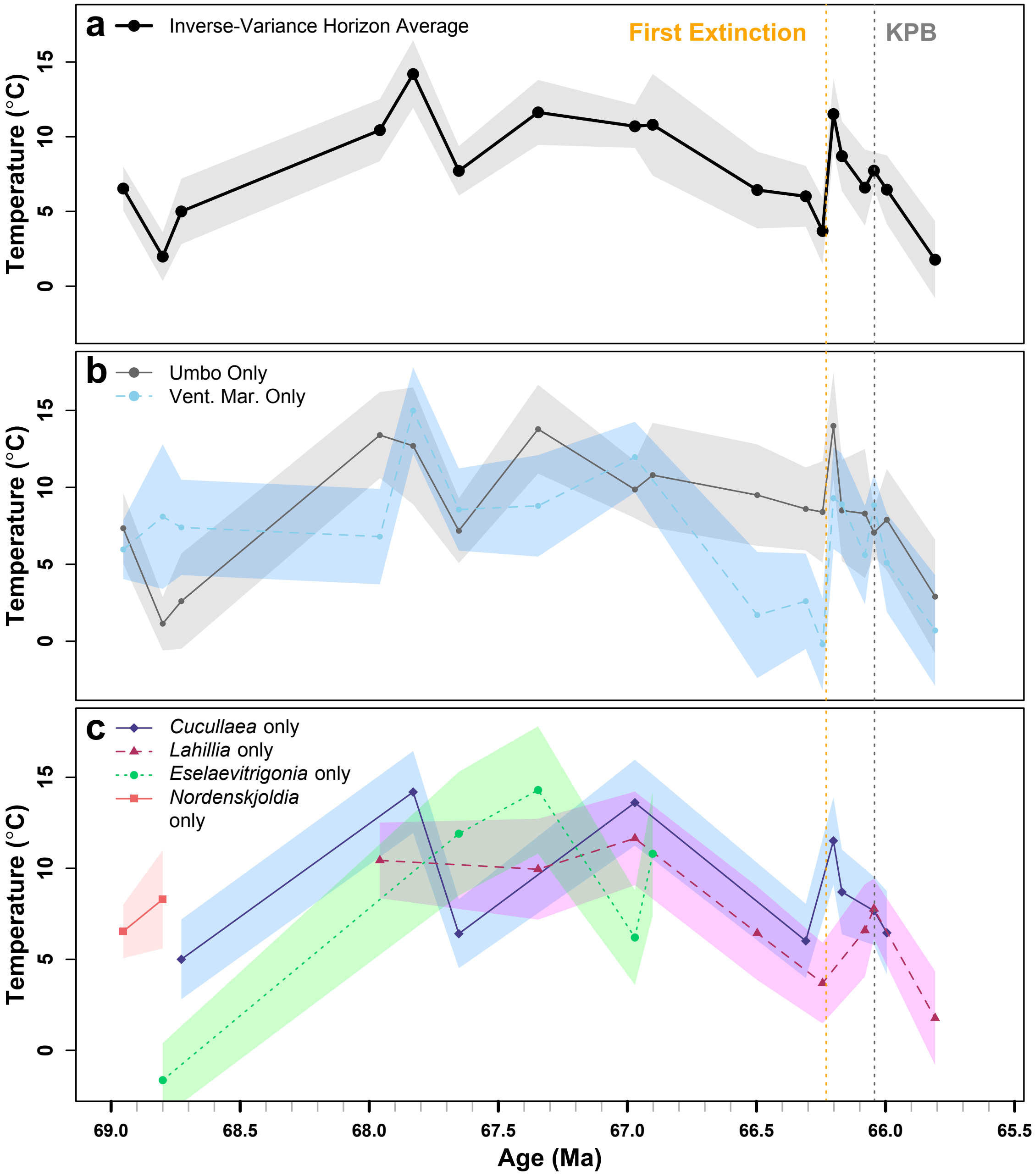

Supplementary Figure 1 | Temperature vs. Age for different subsets of clumped isotope data set. (a) Full composite Δ_{47}-derived temperature record (same as in Fig. 1a). (b) Composite records created by separating data based on shell position. (c) Separated based on species. Horizon means in all three panels were calculated as the inverse variance weighted mean and error, shown as shaded envelope of corresponding color. Samples within ±3.5m stratigraphic position of another sample were combined into a single horizon for horizon means (see Methods). Only *Cucullaea* shows the pre-KPB spike because it is the only species with samples in that stratigraphic interval. Vertical grey dotted line represents the KPg boundary[1] and orange dotted line represents the timing of the first extinction event[2]. Data for this figure can be found in Supplementary Data 6.

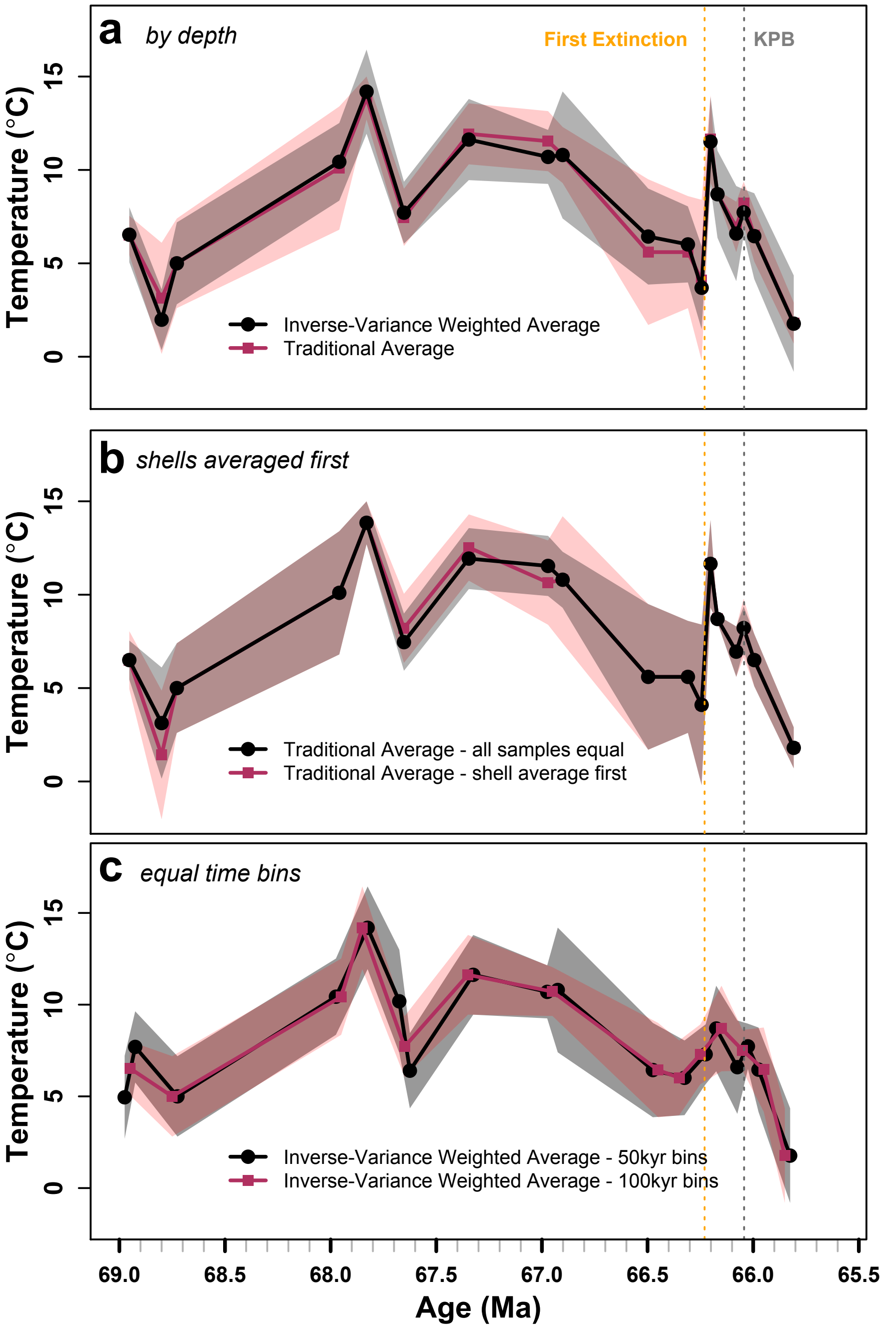

Supplementary Figure 2 | Temperature vs. Age calculated with different averaging methods. (a) Inverse-variance weighted mean (same as in Fig. 1a) compared to traditional mean and 1 SE error. (b) Traditional mean and 1 SE error of all samples in a given horizon compared to taking averages within each shell first, followed by averages of multiple shells. (c) Separated into 50kyr or 100kyr equal time bins. Equal time bins do not capture the full magnitude of either warming pulse due to combining samples from before and after the warming spike into a single bin. Vertical grey dotted line represents the KPg boundary (KPB)[1] and orange dotted line represents the timing of the first extinction event[2]. Data for this figure can be found in Supplementary Data 7 and 8.

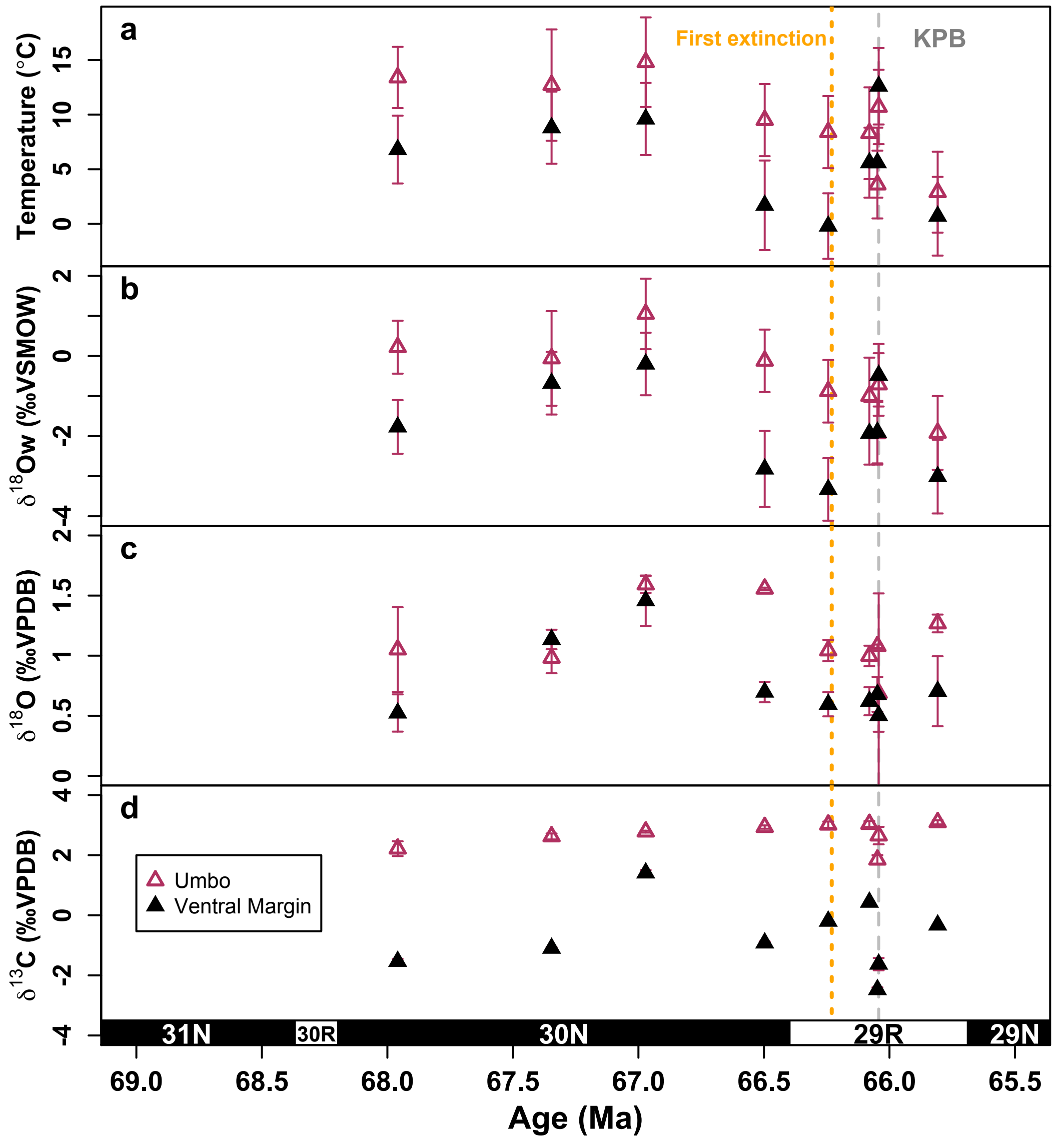

Supplementary Figure 3 | Stable isotope and temperature data for *Lahillia larseni*. (a) Δ_{47}-derived temperature, (b) $\delta^{18}O_w$, (c) carbonate $\delta^{18}O$ and (d) $\delta^{13}C$ vs. Age for *Lahillia larseni*. Error bars are 1 SE for temperature and $\delta^{18}O_w$ and 1 sd for $\delta^{18}O$ and $\delta^{13}C$. *L. larseni* shows consistently lower temperature, lower $\delta^{18}O_w$, lower $\delta^{13}C$, and somewhat lower $\delta^{18}O$ in the ventral margin relative to the umbo position. Vertical grey dashed line represents the KPg boundary[1] and orange dotted line represents the timing of the first extinction event[2]. Data for this figure can be found in Supplementary Data 5.

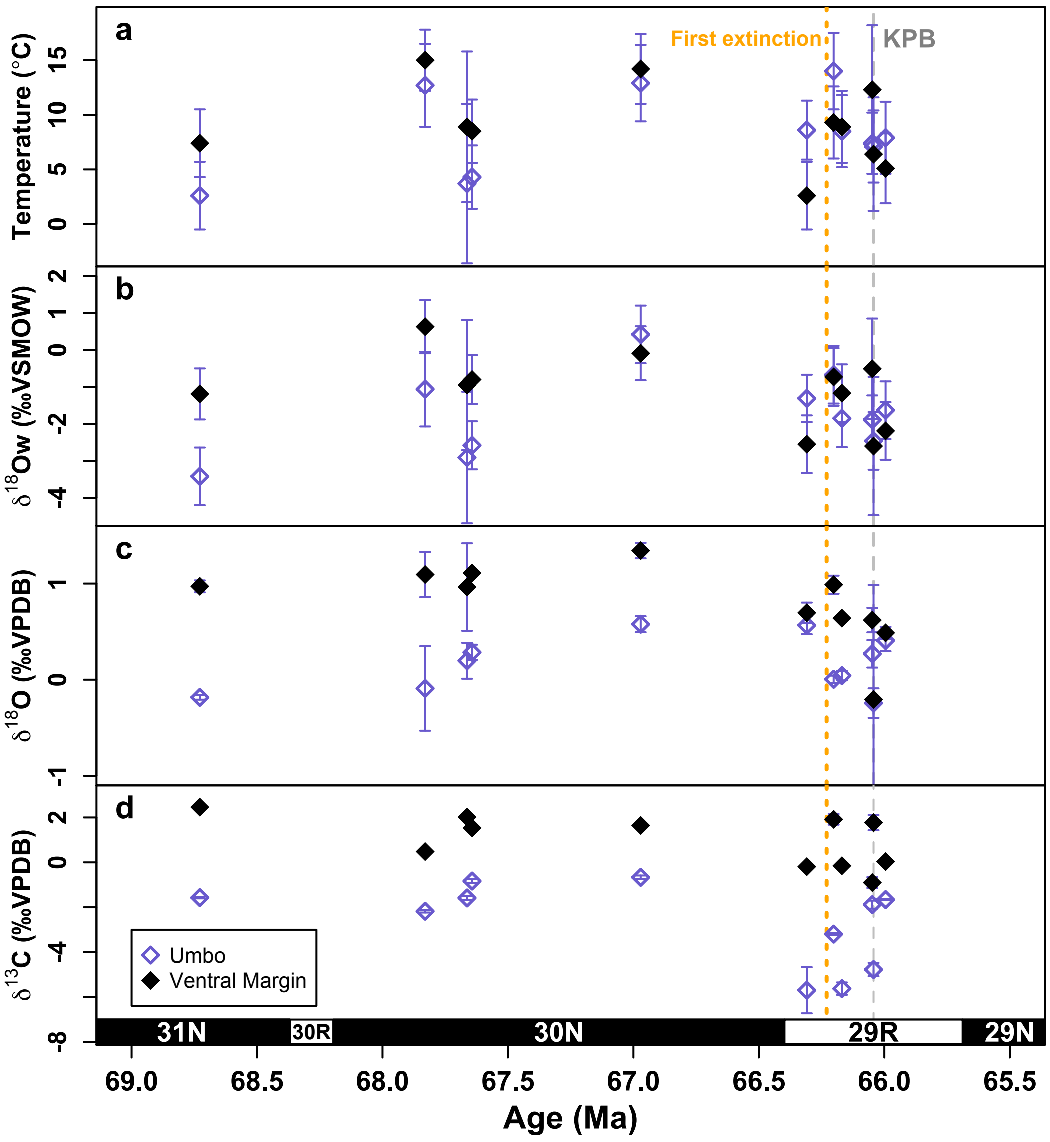

Supplementary Figure 4 | Stable isotope and temperature data for *Cucullaea sp.* (a) Δ_{47}-derived temperature, (b) $\delta^{18}O_w$, (c) carbonate $\delta^{18}O$ and (d) $\delta^{13}C$ vs. Age for *Cucullaea*. Error bars are 1 SE for temperature and $\delta^{18}O_w$ and 1 sd for $\delta^{18}O$ and $\delta^{13}C$. This figure combines *Cucullaea antarctica* and *Cucullaea elliotti.* The four youngest shells are *C. elliotti*. *Cucullaea* shows consistently higher $\delta^{18}O_w$, higher $\delta^{18}O$, and higher $\delta^{13}C$ in the ventral margin relative to the umbo position. Temperature patterns between positions are not as consistent as for other isotopic values. Vertical grey dashed line represents the KPg boundary[1] and orange dotted line represents the timing of the first extinction event[2]. Data for this figure can be found in Supplementary Data 5.

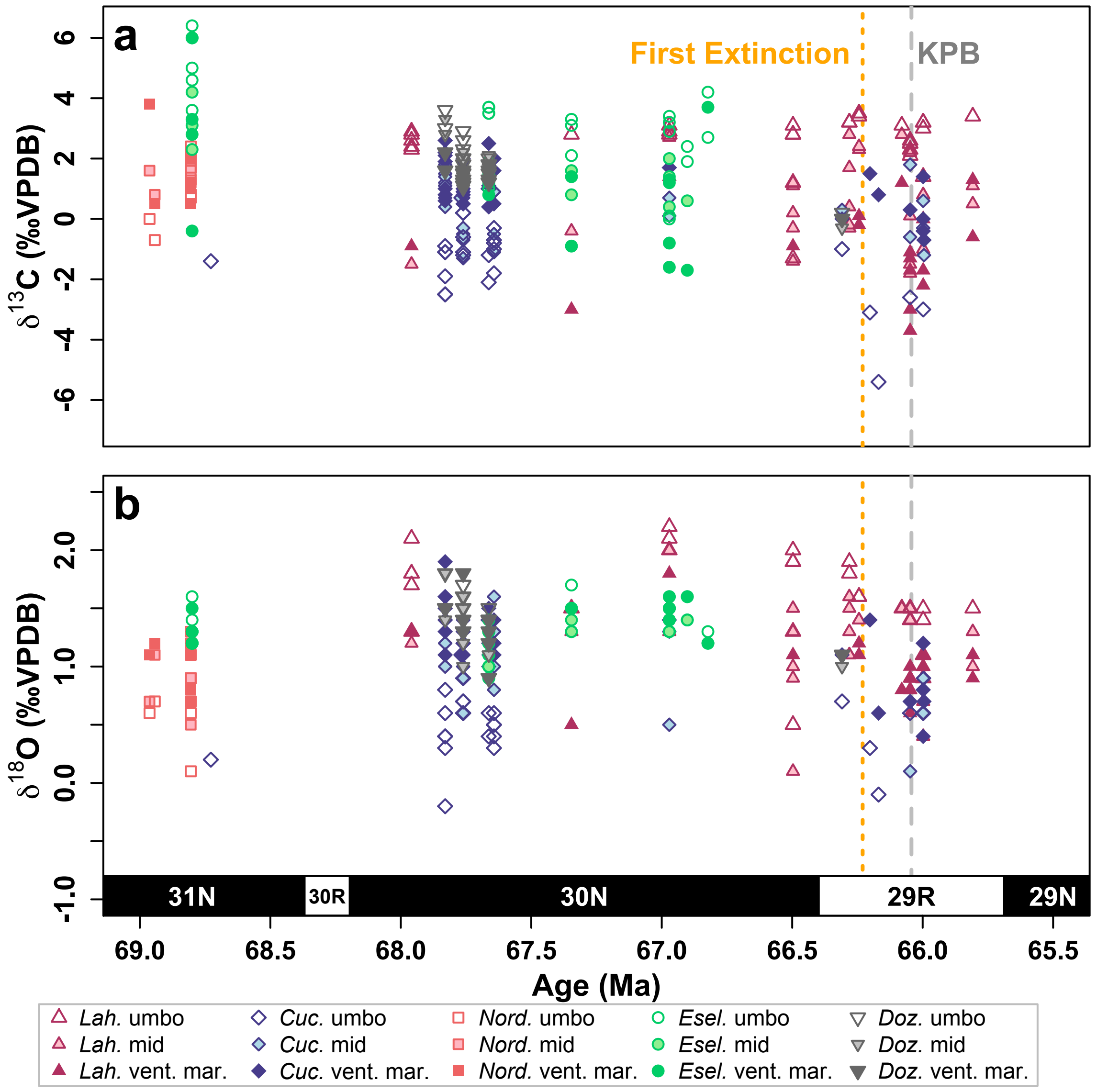

Supplementary Figure 5 | δ^{13}C and δ^{18}O for "full data set". (a) δ^{13}C and (b) δ^{18}O vs. Age, by position, for all samples measured for δ^{13}C and δ^{18}O (the full sample set)[3]. Error on stable isotope measurements was better than 0.1‰ (no error bars shown). δ^{13}C values consistently vary between -2‰ and +2‰ until Chron 29R, where the lowest values are (-6‰). Vertical grey dashed line represents the KPg boundary[1] and orange dotted line represents the timing of the first extinction event[2]. Data for this figure can be found in Supplementary Data 1.

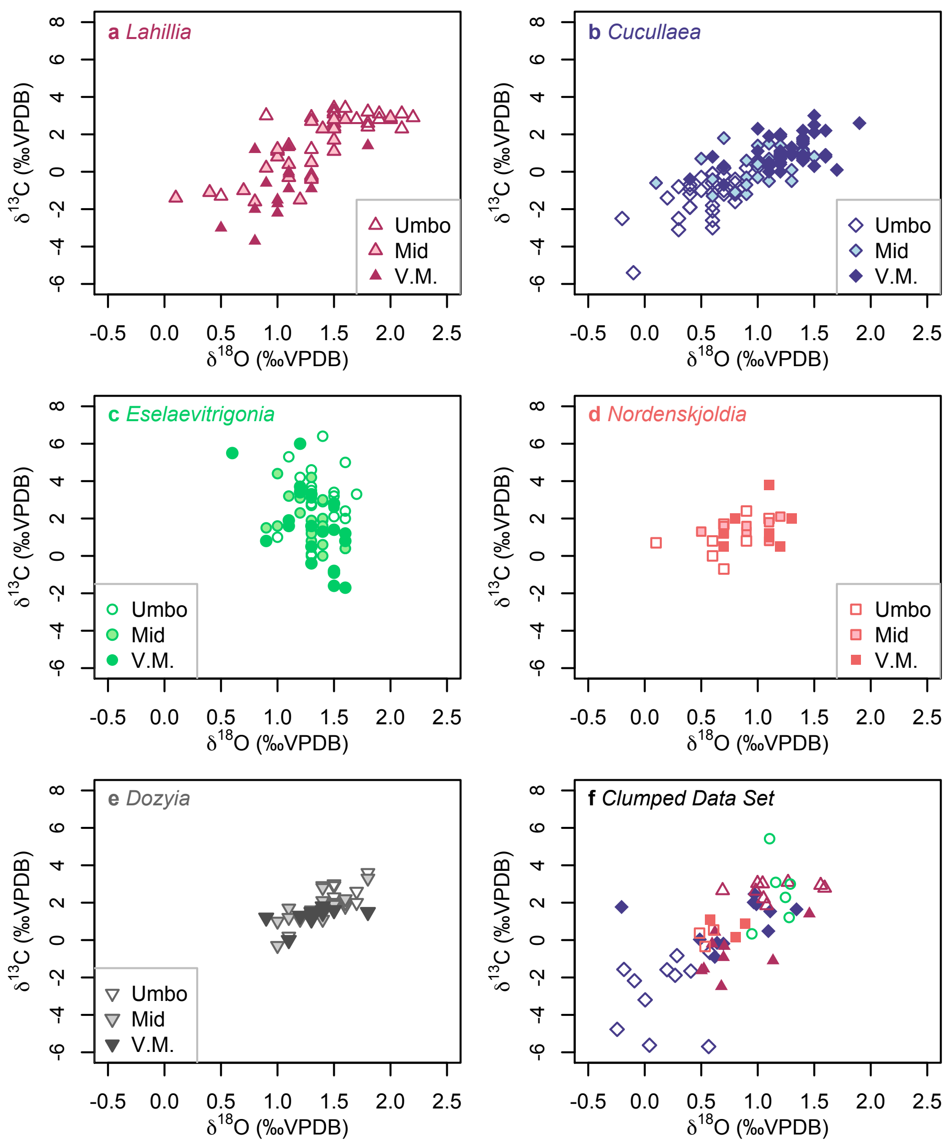

Supplementary Figure 6 | Correlation between δ^{13}C and δ^{18}O, by genera. δ^{18}O vs. δ^{13}C for (a) *Lahillia*, (b) *Cucullaea*, and (c) *Eselaevitrigonia*, (d) *Nordenskjoldia*, and (e) *Dozyia* for the 'full data set'[3], with symbols defining position on shell where sample was drilled. Error on stable isotope measurements was better than 0.1‰ (error bars not shown). (f) shows δ^{18}O vs. δ^{13}C for all samples from the 'clumped data set' for comparison, with the legend found in the other panels. Error on stable isotope measurements made during clumped isotope analysis are on average ~0.15‰ (error bars not shown). *Lahillia* and *Cucullea* show opposite relationships with position, with the heaviest values occurring in the umbo and ventral margin, respectively. *Nordenskjoldia* and *Dozyia* show similar relationships to *Cucullaea* and *Lahillia*, respectively, but of smaller total magnitude. *Eselaevitrigonia* shows no correlation between δ^{18}O and δ^{13}C and shell position. Data for this figure can be found in Supplementary Data 1 for the bulk samples and Supplementary Data 5 for the clumped data set.

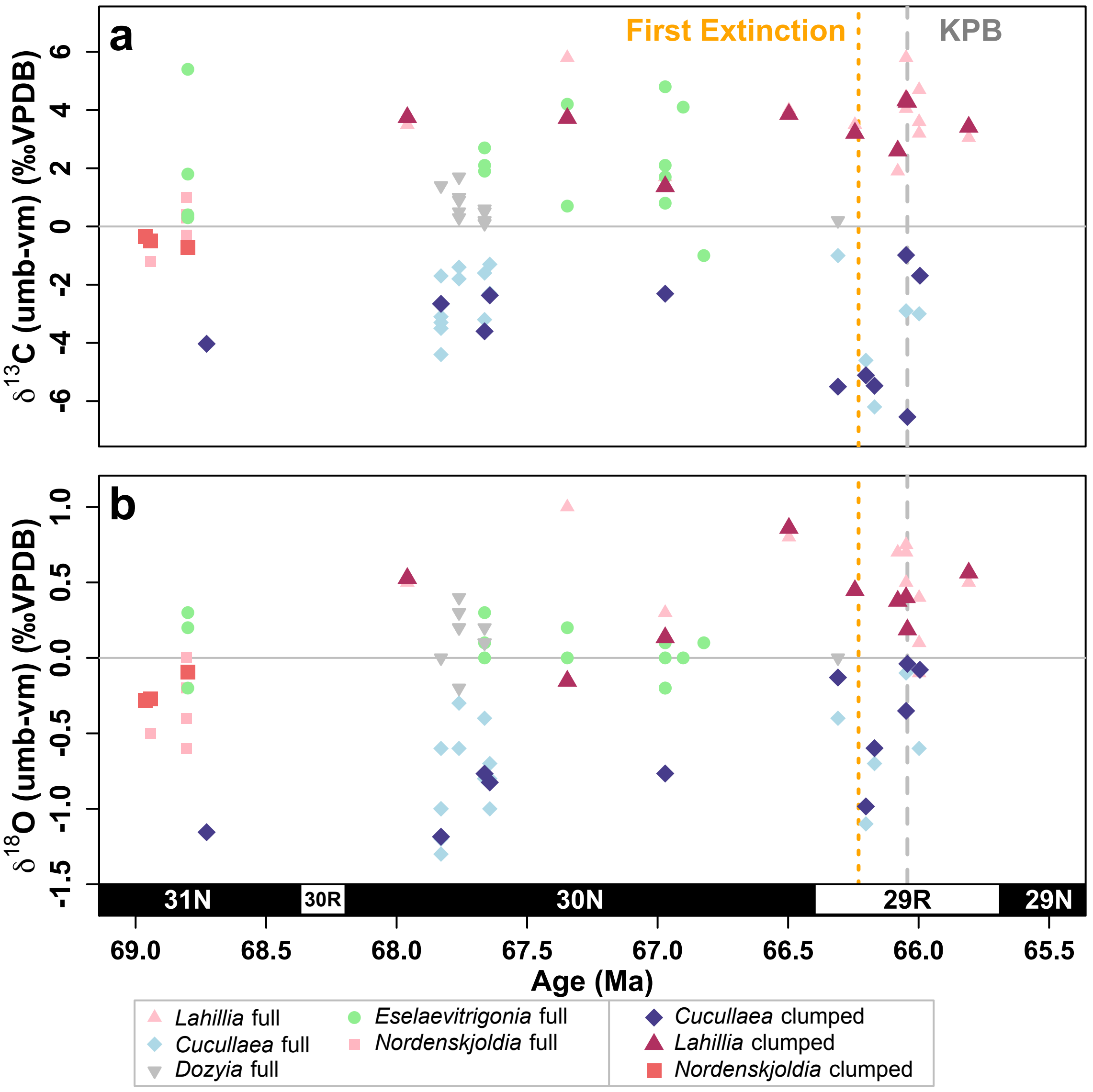

Supplementary Figure 7 | Position-specific differences for full and clumped data sets. Differences in δ^{13}C and δ^{18}O vs. Age for all samples in the full and clumped data sets. All differences calculated as the umbo minus the ventral margin value (umb-vm). The same position-specific patterns by species are seen in the full and clumped data sets, indicating the clumped data set is representative of all samples. Vertical grey dashed line represents the KPg boundary[1] and orange dotted line represents the timing of the first extinction event[2]. Data for this figure can be calculated from data in Supplementary Data 1 for the bulk samples and Supplementary Data 5 for the clumped data set.

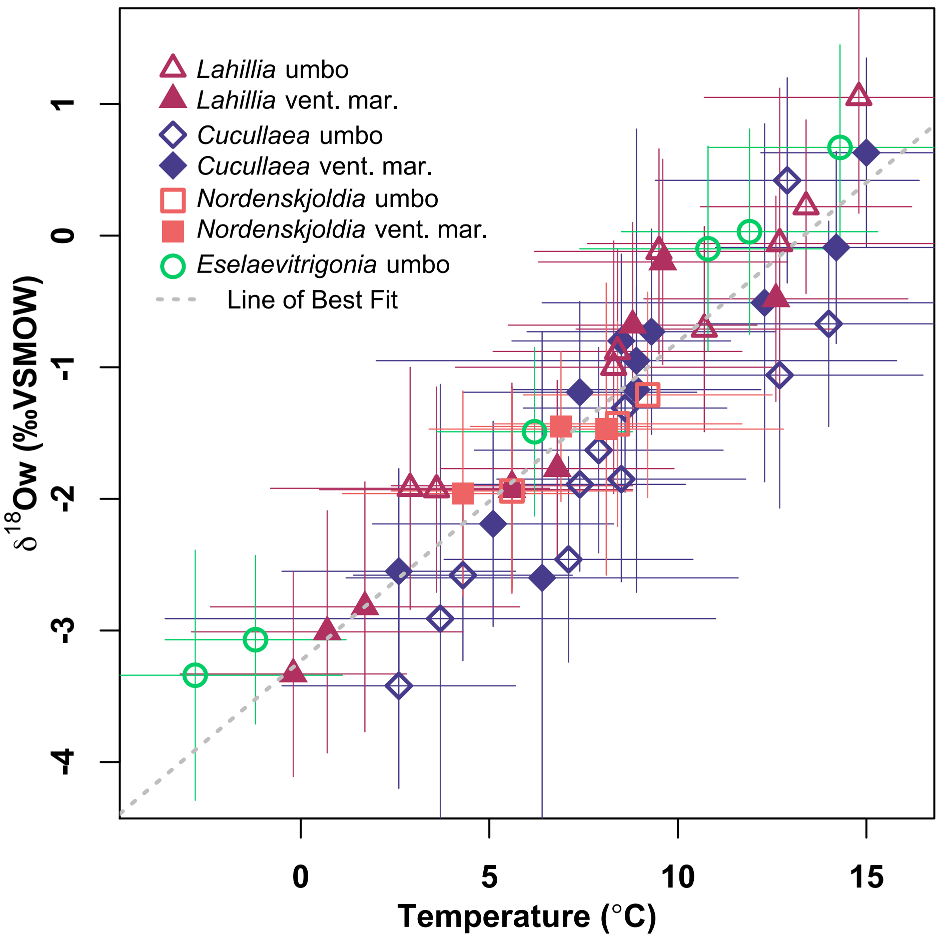

Supplementary Figure 8 | Temperature vs. $\delta^{18}O_w$. Temperature vs. $\delta^{18}O_w$ for all samples measured in the "clumped isotope sample set", showing strong correlation between these two parameters. Error bars represent 1 SE external error. Correlated changes in temperature and $\delta^{18}O_w$ result in intermediate $\delta^{18}O$ values that do not track either parameter (see Fig. 3). The line of best fit (dashed grey) has the equation: $\delta^{18}O_w = 0.242 * \text{Temperature} - 3.230$. This fit has an R^2 value of 0.844 and a p-value of < 2.2E-16. Data for this figure can be found in Supplementary Data 5. See Supplementary Discussion for details about possible causes of this correlation.

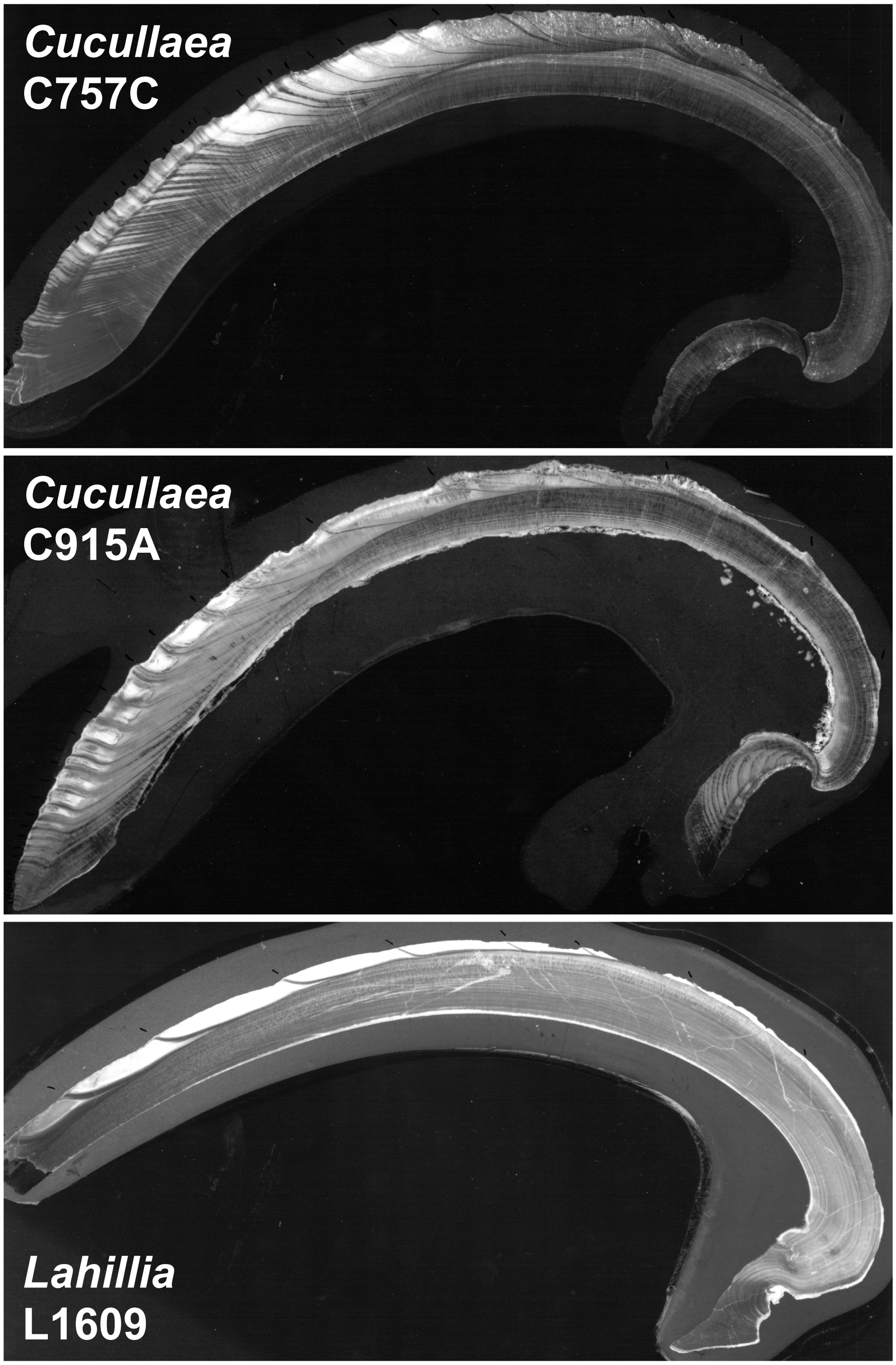

Supplementary Figure 9 | Thin section images. 'Negative images' of representative shell cross sections displaying original growth-band textures. The thin sections were treated as a photographic negative to produce these pictures. Light areas in these photos correspond to the darkest/densest portions of the original shell. For scale, C757C2 is approximately 1cm thick and C915A2 and L757B are approximately 0.5cm thick in the ventral margin area of the shell.

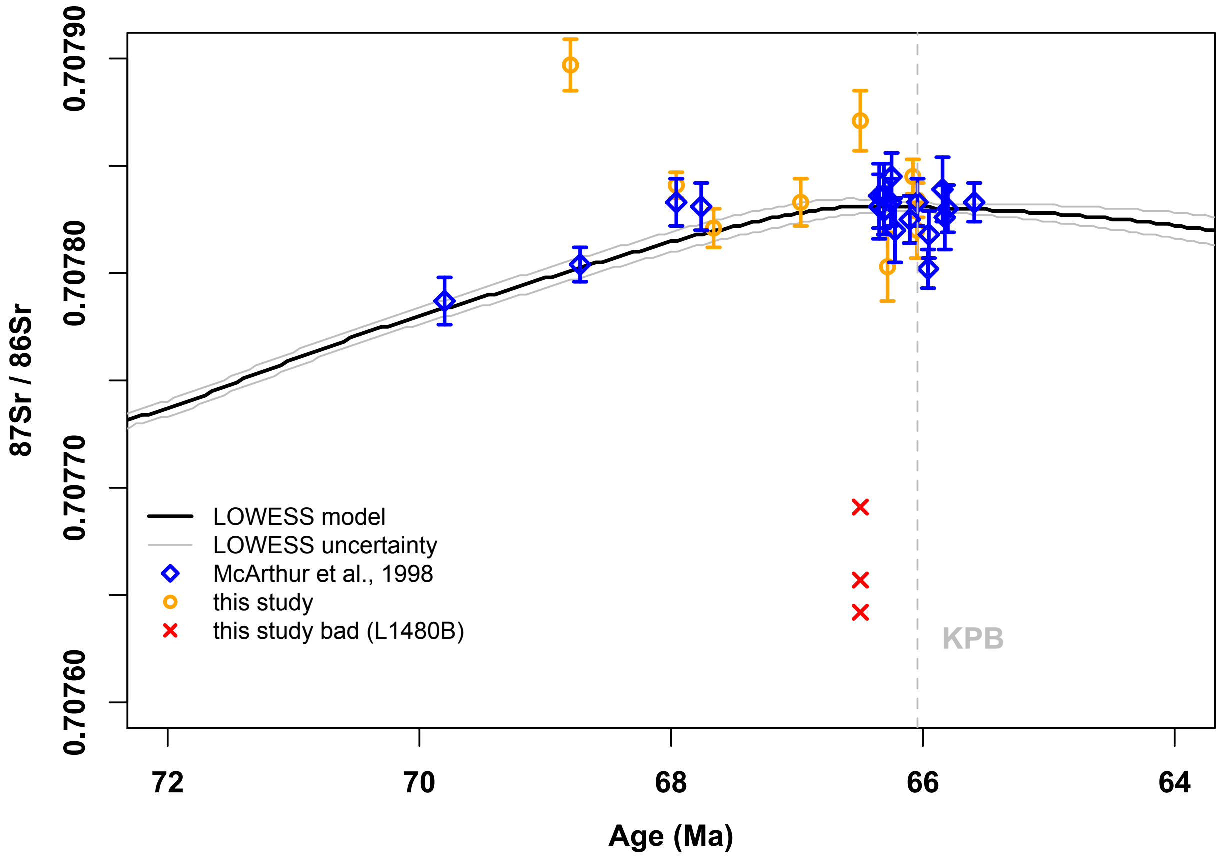

Supplementary Figure 10 | Strontium Isotope data. $^{87}Sr/^{86}Sr$ vs. Age for LOWESS version 5 marine curve[4] and shells measured in this study (orange) and in McArthur *et al.*[5] (blue) plotted with calculated age from age model in this study. Error bars are 2 SE. Sample L1480B, which had anomalous strontium isotopic composition and elevated [Sr] is shown in red x's. Data for this figure are found in Supplementary Tables 2 and 3.

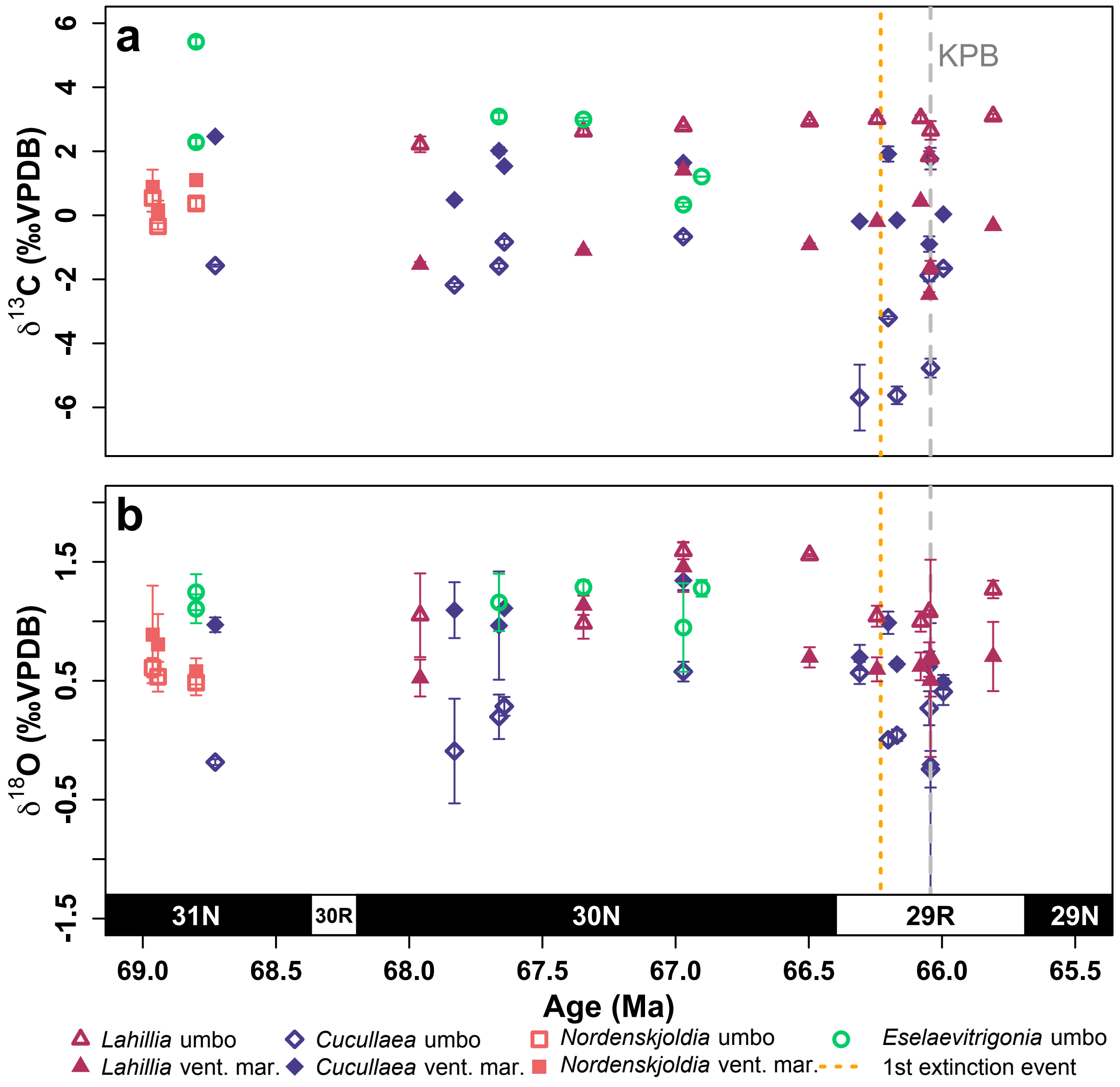

Supplementary Figure 11 | δ^{13}C and δ^{18}O for clumped data set. (a) δ^{13}C and (b) δ^{18}O vs. Age, by position, for all samples in the clumped isotope data set. Error bars are 1 sd. δ^{13}C values consistently vary between -2‰ and +2‰ until Chron 29R, where the lowest values are recorded. Vertical grey dashed line represents the KPg boundary[1] and orange dotted line represents the timing of the first extinction event[2]. Data for this figure can be found in Supplementary Data 5.

Tie Point	Stratigraphic Height (m) (Tobin)	Stratigraphic Height (m) (Zinsmeister)	Age (Ma) (GTS2012)
29R-29N	924	1118	65.688
KPg boundary	865	1059	66.043
30N-29R	789	983	66.398
30R-30N	607	801	68.196
31N-30R	589	783	68.369
31R-31N	401	595	69.269

Supplementary Table 1 | Tie Points used in creation of age model. Position of chron reversals in Tobin framework from supplementary material of Tobin *et al.*[2]. Position of KPg boundary in Zinsmeister section from Zinsmeister[6]. Stratigraphic height of other chron reversal in Zinsmeister framework are calculated as the the position in the Tobin framework plus 194 m (=1059-865). Ages of chron reversals taken from GTS 2012 (ref. 54).

Specimen ID	Strat. Position (m)	Calculated Age	Measured $^{87}Sr/^{86}Sr$	Corrected $^{87}Sr/^{86}Sr$	Analytical Error (±2Se)	Notes
N1624A	692	68.800	0.707897	0.707869	0.000012	
L776E	825	67.959	0.707841	0.707813	0.000006	
C1109D	855	67.663	0.707821	0.707793	0.000009	
L757C	925	66.971	0.707833	0.707805	0.000011	
L1480B	973	66.497	0.707642	0.707614	0.000028	*high [Sr]
L1480B (rerun)	(same)	(same)	0.707657	0.707629	0.000031	*high [Sr]
L1480B (rerun #2)	(same)	(same)	0.707691	0.707663	0.000004	*high [Sr]
L1480E	973	66.497	0.707871	0.707843	0.000014	
L1516A	1008	66.281	0.707803	0.707775	0.000016	
L1161A	1051	66.080	0.707845	0.707817	0.000008	
L477D	1058	66.048	0.707820	0.707792	0.000013	
L1529A	1059	66.043	0.707834	0.707806	0.000008	

Supplementary Table 2 | Strontium isotope data measured in this study. Measured $^{87}Sr/^{86}Sr$ values are corrected based on the difference between NBS987 measured in this study (0.710252 ± 0.000026 (2σ)) and the accepted value of 0.710248. Age is calculated using the magnetostratigraphic age model for this study. Species is defined by the first letter of the sample name (N = *N. nordenskjoldia*, L = *L. larseni*, C = *C. antarctica* in this case).

Zinsmeister Locality #	Stratigraphic Height (plane-projected)	$^{87}Sr/^{86}Sr$	Analytical Error (±2Se)	Calculated Age (from magnetostr.)
768	484	0.707787	0.000011	69.800
772	709	0.707804	0.000008	68.723
776	825	0.707833	0.000011	67.959
1100	845	0.707831	0.000011	67.761
1157	994	0.707831	0.000015	66.347
1157	994	0.707836	0.000015	66.347
1174	1002	0.707829	0.000011	66.309
1174	1002	0.707836	0.000015	66.309
1175	1014	0.707833	0.000011	66.253
774	1015	0.707845	0.000011	66.249
1177	1021	0.707820	0.000015	66.221
1531	1046	0.707825	0.000011	66.104
477	1059	0.707833	0.000011	66.043
1533	1073	0.707802	0.000009	65.959
1534	1074	0.707818	0.000011	65.953
1135	1092	0.707839	0.000015	65.844
1130	1095	0.707826	0.000015	65.826
1537	1099	0.707830	0.000011	65.802
1140	1134	0.707833	0.000009	65.592

Supplementary Table 3 | Strontium isotope data from McArthur *et al.*[5]. Stratigraphic heights have been adjusted based on the plane-projection method[6]. Age calculated based on stratigraphic position using age model of this study.

Supplementary Discussion
Correlation between Temperature and $\delta^{18}O_w$

Supplementary Fig. 8 shows a very strong correlation (R^2 = 0.844, p-value < 2.2E-16) between temperature and $\delta^{18}O_w$ among individual samples. We have interpreted this correlation as reflecting climatologically-controlled, seasonal delivery of continental runoff to the near-shore environment. In colder climates, more snowpack accumulates on land in winter, $\delta^{18}O$ of the snow is lower, and meltwater is delivered in a concentrated spring thaw, reducing near-shore $\delta^{18}O_w$ significantly. In warmer climates, less snowpack accumulates, $\delta^{18}O$ of the snow is higher, and delivery is less concentrated, causing higher near-shore $\delta^{18}O_w$ values than during cold climates. Here we discuss non-environmental ways to produce a correlation between temperature and $\delta^{18}O_w$ and enumerate why the climatological mechanism is the most likely explanation.

One possible explanation for the observed correlation is that variability in Δ_{47}-derived temperature caused by analytical noise or alteration of the clumped isotope signal during sample

drilling, propagated through the $\delta^{18}O$/Temperature/$\delta^{18}O_w$ relationship[7] to produce correlated variability in $\delta^{18}O_w$. With regards to drilling, this preparation method has been used for conventional stable isotope studies for decades and does not affect carbonate $\delta^{18}O$[8]. However, drilling at high speed has the potential to increase the Δ_{47}-derived temperature through frictional heating and resetting. If drilling increased the measured Δ_{47}-derived temperature on a particular sample, it would result in an artificial increase in the calculated $\delta^{18}O_w$ value for that sample. The magnitude of the increase in $\delta^{18}O_w$ would be positively correlated to the magnitude of the increase in temperature according to the $\delta^{18}O$/Temperature/$\delta^{18}O_w$ relationship[7], corresponding to roughly a 0.25‰ increase in $\delta^{18}O_w$ for every 1°C increase in temperature. Similarly, large analytical noise could cause either a positive or negative anomaly in the measured clumped isotope temperature. In this case, the resulting positive or negative perturbation in $\delta^{18}O_w$ would follow the same ratio derived from the $\delta^{18}O$/Temperature/$\delta^{18}O_w$ relationship[7]. If each sample was affected by drilling-induced alteration or analytical noise to a different degree, beginning from a similar starting temperature, the resulting Temperature vs. $\delta^{18}O_w$ scatter plot would show a positive correlation with a slope of ~0.25, similar to Supplementary Fig. 8, which has a slope of 0.242.

There are multiple reasons that suggest the correlation in Supplementary Fig. 8 is not due to drilling-induced alteration. First of all, in our experience, this effect has only been seen at drill speeds much higher than the setting used in this study (15,000 rpm vs. 1,000 rpm used here). Foster *et al.*[8] found 6% conversion to calcite and no change in carbonate $\delta^{18}O$ at drill speeds of ~16,000-21,000 rpm (Δ_{47} was not measured). Although this drilling effect on Δ_{47} has been hypothesized based on a few observations, it has yet to be rigorously analyzed in any peer-reviewed literature. Additionally, the proposed drilling artifact can only increase Δ_{47}-derived temperatures and, therefore, $\delta^{18}O_w$ values. If this were occurring, we would see higher temperatures and $\delta^{18}O_w$ values than expected. Instead, the mean $\delta^{18}O_w$ value for the entire study interval (-1.3±0.8‰ (1sd), mean of horizon averages) agrees with (or is even slightly lower than) the predicted mean value for an ice-free world (-1.0‰) (ref. 9) or for an ice-free world adjusted for the modern latitudinal isotopic gradient (-1.2‰) (ref. 10). Also, the mean ocean temperature for the entire section is 7.7 ± 3.4°C (1sd) (mean of horizon averages), consistent with an independent terrestrial temperature estimate of 7°C from fossil wood[11] and a soil temperature estimate of 10-13°C from branched tetraethers[12]. These observations combine to suggest that broad resetting through drilling has not occurred.

Large analytical noise, which could cause artificial increases or decreases in Δ_{47}-derived temperatures, would be equally likely to affect any given sample. This would result in a larger range in temperatures seen over the study interval, caused by the addition of analytical-noise-induced scatter on top of the 'true' temperature record. If common assumptions about the minimal variability of marine $\delta^{18}O_w$ are to be believed (no variability, constant $\delta^{18}O_w$), the $\delta^{18}O$ data suggests a total temperature range of 8°C (from ~4-12°C). In comparison the clumped isotope record shows a much larger temperature range of ~20°C (from ~-3-15°C), consistent with expectations for the "noise plus 'true' variability" scenario described above. However, in this hypothetical noisy record, there should still remain a correlation between temperature and shell $\delta^{18}O$. In this case, a linear regression between $\delta^{18}O$ and temperature gives an R^2 value of 0.021 – no correlation. R^2 values less than 0.1 are also achieved for all position-species subsets of the data (all *Lahillia* umbo positions, for example) for *Lahillia*, *Cucullaea*, and *Eselaevitrigonia*, with the exception of *Cucullaea* ventral margin, which has an R^2 value of 0.25.

Nordenskjolidia shows R^2 values of 0.67 and 0.80 for the umbo and ventral margin, although this is only based on three points. In fact, for all *Cucullaea* shells, $\delta^{18}O$ values are equal or higher in ventral margin than in the umbo position, suggesting colder temperatures in the ventral margin position. Colder temperatures are also expected for *Lahillia* in its umbo position relative to ventral margin. Clumped isotope temperature reveal the opposite, finding warmer temperatures recorded in *Cucullaea* ventral margin and *Lahillia* umbo, where $\delta^{18}O$ predicted colder. This extreme lack of correlation between $\delta^{18}O$ and temperature goes against the fundamental assumption underlying all $\delta^{18}O$-based paleoclimate studies - that $\delta^{18}O_w$ *should* be fixed, and that, therefore, temperature must be the sole driving force setting $\delta^{18}O$ values – and suggests that $\delta^{18}O$, temperature, and $\delta^{18}O_w$ are related in unusual ways in this data set.

The samples in this study were drilled, prepared, and analyzed over three separate measurement sessions, spanning a total of nine months. The order of analysis of individual replicates was scrambled on a daily, weekly, and monthly basis to avoid potential cumulative effects of measuring the same samples in the same order, or biases caused by unusual measurement conditions on a given day or week. Carbonate standards were continually monitored and showed consistency and reproducibility during the same measurement sessions. These practices are done to reduce biases caused by short- and long- term drift in instrument conditions and make it unlikely that analytical noise would produce artificial spikes or trends in the temperature record that show the agreement between samples through time and the position-specific relationships we observe. Samples of similar stratigraphic age produce similar Δ_{47}-derived temperatures, showing a clear coherence in the temperature record on both short and long timescales. We also see consistent position-specific patterns within species (Fig. 2). For example, all *Lahillia* shells, excluding the two at the KPB, show warmer temperatures in the umbo position relative to the ventral margin. These observations, combined with our laboratory practices designed to avoid bias make it unlikely that analytical noise produced the temperature record we observe.

Another possibility is that the observed correlation is the result of diagenetic alteration occurring in situ on Seymour Island, prior to collection and analysis. For diagenesis to produce the observed correlation between temperature and $\delta^{18}O_w$ but retain the lack of correlation between temperature and carbonate $\delta^{18}O$, alteration must change temperature without changing carbonate $\delta^{18}O$. This could occur by recrystallization in a rock-buffered system or thermal resetting. Our extensive consideration of diagenetic alteration though trace element screening and cathodoluminescence and petrographic observations of textural features, combined with other studies documenting a shallow burial history for Seymour Island[13,14] (see Methods) suggest that this correlation is unlikely to reflect post-depositional alteration in situ.

Temperature is not the dominant control on $\delta^{18}O$ in this data set, yet evidence suggests that the temperature record is robust. Therefore, *the only possible explanation* that fits both the temperature and $\delta^{18}O$ data is that temperature and $\delta^{18}O_w$ are varying in conjunction and in opposition. The combination of the large observed variability in Δ_{47}-derived temperatures (~20°C range) and the small range in $\delta^{18}O$ values forces $\delta^{18}O_w$ to vary significantly (a 5‰ range) and in tight coupling with temperature.

Prior to the development of the clumped isotope paleothermometer, studies universally assumed that $\delta^{18}O_w$ was constant and, therefore, that measured variability in $\delta^{18}O$ was dominated by changes in temperature. As more and more clumped isotope measurements are made and $\delta^{18}O_w$ is directly assessed instead of assumed, we are finding that $\delta^{18}O_w$ doesn't always follow

the invariant behavior we had assumed, and that such changes must be considered when interpreting carbonate $\delta^{18}O$ as a proxy of temperature. In the main text we suggest an environmental explanation for the coupling between temperature and $\delta^{18}O_w$ that we believe is not only plausible, but is the strongest and most parsimonious interpretation of the dataset. We look forward to more studies putting the "fixed $\delta^{18}O_w$" assumption to the test.

Supplementary References:

1. Renne, P. R., *et al*. Time Scales of Critical Events Around the Cretaceous-Paleogene Boundary. *Science* **339,** 684-687 (2013).
2. Tobin, T. S. *et al*. Extinction patterns, $\delta^{18}O$ trends, and magnetostratigraphy from a southern high-latitude Cretaceous-Paleogene section: Links with Deccan volcanism. *Palaeogeogr. Palaeoclimatol. Palaeoecol.* **350**, 180–188 (2012).
3. Dutton, A. Extracting paleoenvironmental records from molluscan carbonate. PhD thesis, University of Michigan (2002).
4. McArthur, J. M., Howarth, R. J. & Shields, G. A. Strontium Isotope Stratigraphy. In *The Geologic Time Scale*, F. M. Gradstein, J. G. Ogg, M. D. Schmitz, G. M. Ogg Eds. (Elsevier, Oxford, 2012), **vol. 1**, chap. 7.
5. McArthur, J. M., Thirwall, M. F., Engkilde, M., Zinsmeister, W. J. & Howarth, R. J. Strontium isotope profiles across K/T boundary sequences in Denmark and Antarctica. *Earth Planet. Sci. Lett.* **160**, 179–192 (1998).
6. Zinsmeister, W. J. Late Maastrichtian short-term biotic events on Seymour Island, Antarctic Peninsula. *J. Geol.* **109**, 213–229 (2001).
7. Kim, S. T., O'Neil, J. R., Hillaire-Marcel, C. & Mucci, A. Oxygen isotope fractionation between synthetic aragonite and water: Influence of temperature and Mg^{2+} concentration. *Geochim. Cosmochim. Acta*, **71**, 4704-4715 (2007).
8. Foster, L. C., Andersson, C., Hoie, H., Allison, N., Finch, A. A., & Johansen, T. Effects of micromilling on $\delta^{18}O$ in biogenic aragonite. *Geochem. Geophys. Geosyst.*, **9**, Q04013 (2008).
9. Shackleton, N. J. & Kennett, J. P. Paleotemperature history of the Cenozoic and the initiation of Antarctic glaciation: oxygen and carbon isotope analyses in DSDP Sites 277, 279, and 281. In *Proceedings of the Ocean Drilling Program, Initial Results* **29**, 743-755 (1975).
10. Zachos, J. C., Stott, L. D. & Lohmann, K. C. Evolution of Early Cenozoic marine temperatures. *Paleoceanography* **9**, 353–387 (1994).
11. Francis, J. E. & Poole, I. Cretaceous and early Tertiary climates of Antarctica: evidence from fossil wood. *Palaeogeogr. Palaeoclimatol. Palaeoecol.* **182**, 47–64 (2002).
12. Kemp, D. B. *et al.* A cool temperature climate on the Antarctic Peninsula through the latest Cretaceous to early Paleogene. *Geology* **42**, 583–586 (2014).
13. Pirrie, D., Ditchfield, P. W. & Marshall, J. D. Burial Diagenesis and Pore-fluid Evolution in a Mesozoic Back-Arc Basin: The Marambio Group, Vega Island, Antarctica. *J. Sed. Rsch.* **A64** (3), 541-552 (1994).
14. Palamarczuk, S., Ambrosini, G., Villar, H., Medina, F., Martinez Macchiavello, J. C., & Rinaldi, C. Las Formaciones López de Bertodano y Sobral en la Isla Vicecomodoro Marambio, Antártida: IX Congreso Geológico Argentino, *Actas*, **1**, 399-419 (1984).
